# JUN dependency in distinct early and late BRAF inhibition adaptation states of melanoma

**DOI:** 10.1038/celldisc.2016.28

**Published:** 2016-09-06

**Authors:** Bjoern Titz, Anastasia Lomova, Allison Le, Willy Hugo, Xiangju Kong, Johanna ten Hoeve, Michael Friedman, Hubing Shi, Gatien Moriceau, Chunying Song, Aayoung Hong, Mohammad Atefi, Richard Li, Evangelia Komisopoulou, Antoni Ribas, Roger S Lo, Thomas G Graeber

**Affiliations:** 1 Department of Molecular and Medical Pharmacology, University of California Los Angeles, Los Angeles, CA, USA; 2 Crump Institute for Molecular Imaging, University of California Los Angeles, Los Angeles, CA, USA; 3 Division of Dermatology, Department of Medicine, University of California Los Angeles, Los Angeles, CA, USA; 4 Division of Hematology-Oncology, Department of Medicine, University of California Los Angeles, Los Angeles, CA, USA; 5 Division of Surgical-Oncology, Department of Surgery, University of California Los Angeles, Los Angeles, CA, USA; 6 Jonsson Comprehensive Cancer Center, University of California Los Angeles, Los Angeles, CA, USA; 7 California NanoSystems Institute, University of California Los Angeles, Los Angeles, CA, USA

**Keywords:** Drug resistance, BRAF inhibition, EMT, melanoma, phospho-proteomics

## Abstract

A prominent mechanism of acquired resistance to BRAF inhibitors in *BRAF*^*V600*^-mutant melanoma is associated with the upregulation of receptor tyrosine kinases. Evidences suggested that this resistance mechanism is part of a more complex cellular adaptation process. Using an integrative strategy, we found this mechanism to invoke extensive transcriptomic, (phospho-) proteomic and phenotypic alterations that accompany a cellular transition to a de-differentiated, mesenchymal and invasive state. Even short-term BRAF-inhibitor exposure leads to an early adaptive, differentiation state change—characterized by a slow-cycling, persistent state. The early persistent state is distinct from the late proliferative, resistant state. However, both differentiation states share common signaling alterations including JUN upregulation. Motivated by the similarities, we found that co-targeting of BRAF and JUN is synergistic in killing fully resistant cells; and when used up-front, co-targeting substantially impairs the formation of the persistent subpopulation. We confirmed that JUN upregulation is a common response to BRAF inhibitor treatment in clinically treated patient tumors. Our findings demonstrate that events shared between early- and late-adaptation states provide candidate up-front co-treatment targets.

## Introduction

Advanced metastatic melanoma is a cancer with traditionally poor treatment options [1]. However, in 2002, BRAF^V600E^ was discovered as a common melanoma oncogene that is detected in approximately 50% of patients [2, 3]. This led to the development and eventual FDA approval of the BRAF inhibitor vemurafenib/PLX4032 and dabrafenib/GSK2118436 for patients with advanced melanoma [4–7]. These BRAF inhibitors induce an objective response rate beyond 50% but are limited by a median duration of response of 6–7 months [6]. As with other cancer therapies, intrinsic, adaptive and acquired resistance pose major challenges toward further clinical benefits of BRAF inhibitors [8].

The various reported BRAF inhibitor resistance mechanisms help to explain the continuum of evolutionary processes underlying intrinsic, adaptive and acquired resistance. These include genetic alterations that reactivate MAPK signaling such as NRAS mutations [9], MEK mutations [10] or mutant BRAF amplification [11], which clearly account for acquired or late clinical resistance [12]. Other resistance mechanisms that involve upregulation of receptor tyrosine kinases (RTKs) and a potential rebalancing of signaling toward the PI3K/AKT axis likely account for early resistance associated with intrinsic or adaptive processes [12]. These RTK alterations include IGF1R activation [13], overexpression of PDGFR-beta (PDGFRB), EGFR [12, 14] and potentially other RTKs (RTK-associated resistance mechanism; [9, 14–16]). In addition, melanoma cells can show an early adaptive response to vemurafenib treatment with subpopulations undergoing cell death induction or growth arrest [17, 18], preceding proliferative resistance. This persistent survival state capable of later proliferation may function as a reservoir for late, acquired resistance formation driven by genetic selection [12].

For other cancer types, systems-wide resistance programs have been identified, which reflect complex signaling and phenotypic alterations accounting for intrinsic or adaptive drug resistance. Most prominently, the transition from an epithelial to a mesenchymal differentiation state (EMT) has been reported for various cancers [19–21]. Although mainly thought to provide cancer cells with a more invasive phenotype, EMT has been also directly linked to cancer drug resistance [22–27]. An EMT-like transition has been reported to contribute to melanoma metastasis [28] and, in general, melanoma cells show a high level of differentiation state plasticity [29–32]. Initial reports on the RTK-resistance mechanism implicated upregulation of RTKs such as PDGFRB, IGF1R and MET [9, 13, 33]. However, a more complex cellular adaptation to vemurafenib treatment was indicated both by these initial studies and by subsequent reports [34, 35]. For instance, other RTKs such as EGFR and AXL are also upregulated [36, 37], upregulation of RTKs shows temporal dynamics, and pharmacological inhibition of single RTKs, such as PDGFRB, is not fully effective in impeding resistance. Moreover, the resistant cells show morphological alterations such as a flatter and fibroblast-like appearance. These spindled and proliferative resistant melanoma cells are often found to be derived from short-term adapted, persisted cells that survived the stress of BRAF inhibition.

Here using (phospho-) proteomic profiling, we further characterize the extent to which the observed temporal dynamic of RTK upregulation, morphologic switches and cell cycle adaptations are components of a complex and large-scale adaptation program and investigate the evolution of these resistant cells using an integrative proteomic and genomic approach. In systems-level comparisons, we find the early- and late-adaptive states to be distinct, but to share common signaling alterations. Motivated by the similarities, we demonstrate that targeting an event shared between the adaptation states, JUN upregulation, can provide an effective up-front co-treatment strategy against the adaptive response.

## Results

### RTK-associated resistance displays extensive proteomic alterations in cell attachment and cytoskeleton signaling that are associated with a more mesenchymal state

**Figure 1:**
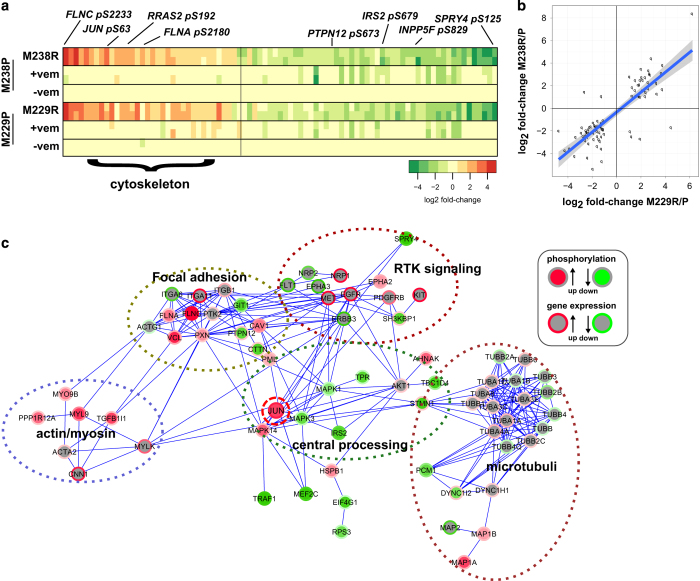
Proteomic analysis of the global signaling state of melanoma cells with RTK-resistance mechanism reveals extensive alterations in cell attachment and cytoskeleton-related signaling processes. (**a**) Global phospho-profiling of two parental (P)/resistant (R) cell line pairs of the RTK-resistance mechanism (M229P/R and M238P/R; vem=6 h treatment with 1 μm vemurafenib/PLX4032 (vem)). Each column of the heatmap represents a phospho-peptide. The fold change of phosphorylation compared with the respective parental cell line is color coded in shades of red (upregulated in resistant cells) or green (downregulated in resistant cells). The phospho-profile was generated with a quantitative, label-free mass-spectrometry technique established in our laboratory [38]. (**b**) Significantly altered phosphorylation sites show same trend in both cell line pairs. A highly significant association between the direction of change for the M229 and M238 pair is observed (Fisher’s exact test; *P*-value <2.2×10^−16^). See Supplementary Figure S3 for more details. (**c**) Illustrative functional protein network of differentially regulated phospho-proteins highlights the observed alterations in cytoskeleton and cell attachment processes in RTK-resistance mechanism cells. The node core and border color shows phosphorylation and gene expression regulation (see legend; darker shades indicate stronger deregulation; missing values are gray). Gene expression data are from Nazarian *et al.* ([9]; geometric mean of fold changes for M238R/P and M229R/P). The network was generated with the Reactome FI plugin in Cytoscape [94]. The Reactome FI database combines curated interactions from Reactome and other pathway databases with statistically filtered uncurated pairwise relationships from various sources including protein interaction and gene co-expression data sets. Phospho-proteins significantly altered in RTK-resistance mechanism supplemented with differentially regulated genes were linked by functional interactions from the Reactome FI database (network edges). The clusters were identified manually, guided by computational analysis [95].

To gain further insights into a previously reported BRAF inhibitor resistance mechanism that involves upregulation of RTKs including PDGFRB (RTK-associated resistance mechanism; [9]), we compared the signaling network states of two melanoma cell subline pairs of parental (P) and derived resistant (R) cells (M229P/R and M238P/R). We used previously established and validated, quantitative, mass-spectrometry-based phospho-profiling procedures [38–41]. First, we focused on phospho-tyrosine signaling using a pan-specific anti-phospho-tyrosine enrichment procedure (Supplementary Figure S1 and Supplementary Table S1). We only identified a limited number of significantly altered tyrosine-phosphorylation sites, which included upregulated phosphorylation of focal adhesion components (paxillin (PXN)) and caveolin (CAV1) in the resistant cells. The lack of a more global increase in phospho-Tyr signaling was initially unexpected for an RTK-associated resistance mechanism, but confirmed by western blot (Supplementary Figure S2), and further motivated a more global analysis of this resistance mechanism. For this, we used a complementary phospho-profiling procedure, in which titania material enriches for peptides phosphorylated on serine, threonine or tyrosine (Figure 1a, Supplementary Figure S1, Supplementary Table S2). Importantly, both cell line pairs showed high correspondence between the observed signaling alterations (Figure 1b, Supplementary Figure S3). Upregulated signaling events in the resistant cells included phosphorylation of JUN (pS63; [42]), and phosphorylation of the RAS-family member RRAS2 (pS192). Signaling events upregulated in resistant cells were significantly enriched for cytoskeletal components (Figure 1a, Supplementary Table S2). Visualization of a functional interaction network for the observed signaling alterations highlights several perturbed signaling processes, most prominent of which included signaling perturbations in cytoskeleton and cell attachment processes (Figure 1c).

**Figure 2:**
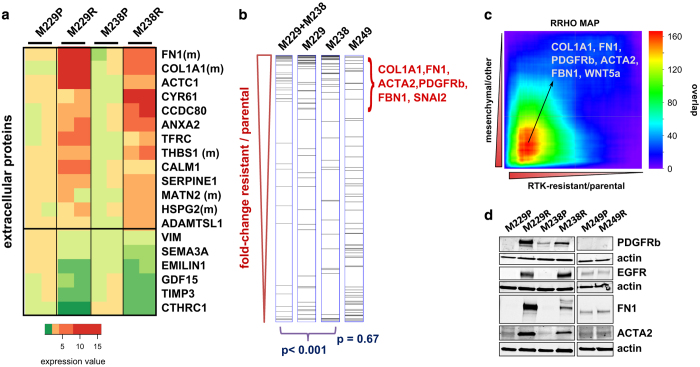
RTK-resistance mechanism melanoma cells are shifted to a mesenchymal de-differentiation state. (**a**) Proteomic profiling of the extracellular matrix (ECM) components of two parental/resistant cell line pairs with RTK upregulation. Heatmap representation of significantly altered ECM components (false discovery rate <0.1) ordered by the signed *t*-test of differential expression between resistant and parental cells. The color-coded ‘expression values’ were calculated as the median of all mean-centered peptide quantification values for a given protein (see 'Materials and Methods' section). Proteins labeled with ‘m’ have been associated with mesenchymal expression, previously (see the main text). (**b**) Upregulated genes in RTK-resistant melanoma cells (M229R, M238R) are enriched for mesenchymal signature genes. Gene expression signatures ranked by fold change for two parental/resistant cell line pairs with RTK upregulation (M229R/P, M238R/P and their sum) were assessed for the enrichment of mesenchymal signature genes (identified by Cheng *et al*. [47]). Both RTK-upregulated resistant cells (M229R and M238R), but not the NRAS-mutated resistant cell line (M249R), show significant enrichment of mesenchymal signature genes among the overexpressed genes (*P*<0.001). (**c**) Direct comparison of RTK-upregulated resistant cell signature (log fold change sum of M229R/P+M238R/P) with mesenchymal (stem cell) signature derived from a global gene expression data set [50]. Comparison with the rank–rank hypergeometric overlap (RRHO) algorithm [92] shows a significant overlap between genes upregulated both by the resistant melanoma cells and by mesenchymal cells. (**d**) Western blot for expression of select marker genes. M249P and M249R cells (NRAS-resistance mechanism) were included for comparison. See Supplementary Figure S4 for additional comparisons between the RTK and NRAS mechanisms. RTK, receptor tyrosine kinase.

To investigate whether the observed global alterations in cell attachment processes are accompanied by alterations in the extracellular context of these cells, we compared the extracellular matrix (ECM) secreted by the two parental/resistant cell line pairs (Figure 2a, Supplementary Table S3). The ECM of the resistant cells showed a striking increase in collagen alpha-1 chain type I (COL1A1), fibronectin (FN1), thrombospondin 1 (THBS1), perlecan (HSPG2) and matrilin 2 (MATN2). Interestingly, all five proteins have been previously associated with a mesenchymal differentiation state [43–45]. Moreover, PDGFRB expression itself, the initial hallmark of the analyzed resistance mechanism, is a marker for mesenchymal cells [46].

These findings, together with the previously noted morphological changes of the resistant cells (flatter, larger and more fibroblast-like; [9]), motivated us to further test whether the RTK-resistance mechanism is associated with a differentiation state change to a more mesenchymal state. To this end, we evaluated more systematically the expression pattern of mesenchymal marker genes in the resistant cells (Figure 2b; [47]). Both RTK-resistance mechanism cell lines (M229R and M238R), but not an NRAS-resistance (MAPK reactivation) mechanism cell line (M249R), demonstrate a significant upregulation of mesenchymal marker genes including *collagen alpha-1 chain type I* (*COL1A1*), *fibronectin* (*FN1*) and *alpha smooth muscle actin* (*ACTA2*). This is further corroborated by the significant enrichment of ‘mesenchymal’ gene sets from the CCancer database in the RTK-mechanism gene expression signature (Supplementary Table S4; [48, 49]). Furthermore, we directly compared the RTK-resistance mechanism gene expression signature with a mesenchymal expression signature from a published global map of human gene expression (Figure 2c; [50]). Again, a highly significant overlap of the genes upregulated in the RTK-mechanism signature and the mesenchymal signature genes supports that the resistant melanoma cells are in a state that strongly resembles mesenchymal cells. Finally, to further validate the differentiation state change of the RTK-resistant cells toward a more mesenchymal state, we confirmed the expression of select marker genes (PDGFRB, FN1 and ACTA2) at the protein level (Figure 2d).

### RTK-resistance mechanism resembles the invasive phenotype associated with a common de-differentiation state in melanoma

**Figure 3:**
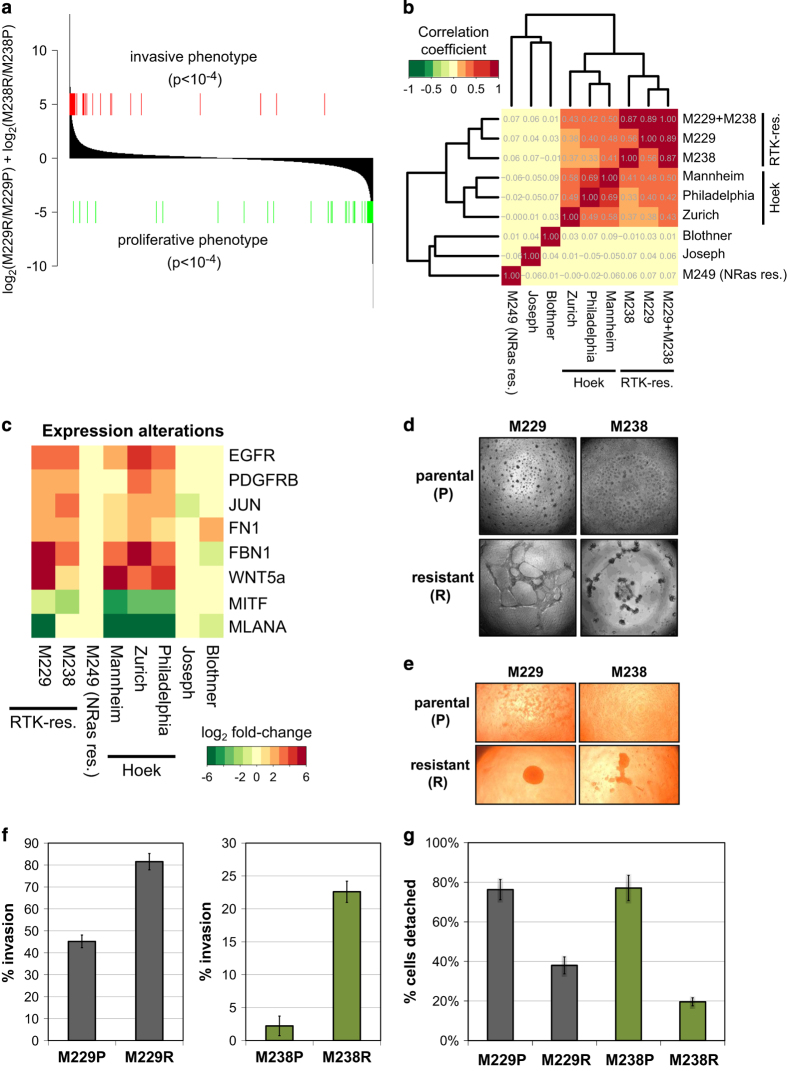
The RTK-resistance de-differentiation mechanism closely resembles the melanoma phenotypic switch. (**a**) Significant similarity of the Hoek–Dummer and RTK-mechanism gene expression signatures. Differentially expressed genes in cell lines with RTK-mechanism of resistance (M229R and M238R)—vs their parental lines (M229P and M238P)—were ranked by their sum fold change difference (black bars). The locations of genes upregulated (red lines, ‘invasive phenotype’) or downregulated (green lines, ‘proliferative phenotype’) by the Hoek–Dummer switch phenomenon (invasive phenotype) are indicated. A significant enrichment of the Hoek–Dummer signature genes is seen at the far ends of the RTK-mechanism distribution (permutation *P*-value <10–4). (**b**) Clustered correlation matrix shows similarity of RTK-mechanism cells and ‘invasive phenotype’ cells (Hoek–Dummer switch phenomenon). Pairwise Pearson correlation coefficients were calculated for nine gene signatures and clustered using a hierarchical clustering procedure. These included three RTK-mechanism-related signatures (M229 (resistant vs parental), M238 (resistant vs parental), M229+M238 (both RTK-mechanism signatures combined)), three different phenotypic switch signatures (Mannheim, Philadelphia and Zurich; ‘invasive’ vs ‘proliferative’ phenotype; [4]), and three control signatures (Blothner (CDKN2A status); [25]), Joseph (PLX4032 treatment; [26]), and M249 (NRAS-based resistance mechanism; [2]). (**c**) Selected expression differences for the RTK-mechanism, phenotypic switch and control gene signatures. The log_2_ fold change difference is color coded (red, upregulated in resistant or invasive cells; green, downregulated). (**d**) Both parental/resistant cell line pairs show a phenotypic switch in the tubuli-formation assay. (**e**) The resistant cells form a dense cell cluster in a sphere formation assay. (**f**) Resistant cells show increased migration through Matrigel in a transwell invasion assay. (**g**) Resistant cells demonstrate slower detachment when treated with trypsin/EDTA (percent detached after 3 min). EDTA, ethylenediaminetetraacetic acid; RTK, receptor tyrosine kinase.

Having established that the RTK-resistance mechanism cells have undergone a transition to a more mesenchymal state, which is reminiscent of an epithelial to mesenchymal transition in other cancer types, we investigated the relation of this process with known melanoma biology. On the basis of overlapping marker genes and proteins (for example, FN1 upregulation and MITF downregulation), we identified a biological similarity to a reported phenotypic switch in (BRAF inhibitor-naive) melanoma, as reported in a series of publications by Hoek, Dummer and colleagues. Here, melanoma cells transition between a ‘proliferative’ and an ‘invasive’ state [29–32]. Notably, the gene expression signature of the RTK-resistance mechanism highly overlaps with the ‘invasive phenotype’ signature (Figure 3a; [30]). This was further confirmed by a more extensive pairwise rank-based correlation analysis of RTK-mechanism, phenotypic switch and negative control signatures (Figure 3b). This similarity is also apparent on the level of individual marker genes (Figure 3c). Note that upregulation of RTKs (EGFR and PDGFRB) is a component of the ‘invasive’ phenotype signature. Thus, these two transition processes—resistance formation upon exposure to BRAF inhibitors and the more stochastic melanoma phenotypic switch—highly overlap.

These gene expression similarities predict similarities in the resulting phenotypes. Phenotypes reported for the ‘invasive’ state include increased invasion and formation of tubuli-like structures on Matrigel and other alterations in cell attachment and migration processes [30, 31]. We tested these phenotypes for the two cell line pairs and found similar phenotypic alterations for the RTK-resistance mechanism cells: increased tubuli-like structure formation on Matrigel (Figure 3d), increased cellular clustering/sphere formation (Figure 3e), increased invasion through Matrigel (Figure 3f) and stronger attachment (Figure 3g). In contrast, the NRAS-mechanism M249 parental/resistant cell line pair does not show a corresponding increase in mesenchymal markers including FN1, ACTA2 and PDGFRB nor an induction of tubuli-like structure formation (Figure 2d, Figure 3c and Supplementary Figure S4). Hence, the RTK-associated resistance mechanism involves an extensive differentiation state switch to a more mesenchymal and ‘invasive’ state.

### BRAF inhibition-induced early adaption persistent cells demonstrate a distinct de-differentiation state compared with long-term adaptation

**Figure 4:**
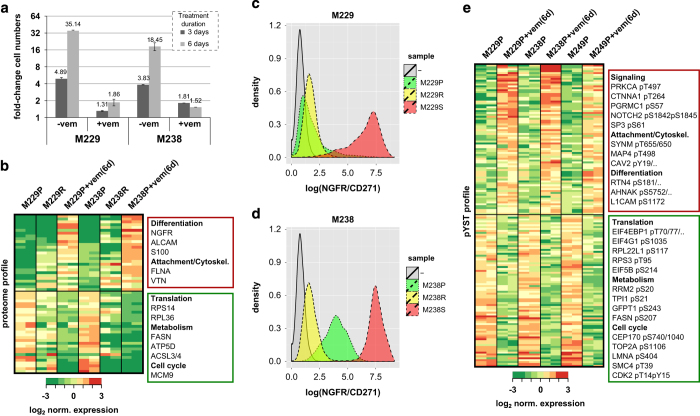
BRAF inhibition-induced early adaption persistent cells demonstrate a distinct de-differentiation state compared with long-term adaptation. (**a**) Vemurafenib treatment results in a persistent, viable but growth-arrested cell population. Two melanoma cell lines (M229P and M238P) were cultured with and without 1μm vemurafenib/PLX4032 (PLX). After 3 and 6 days, the number of viable cells was counted and compared with the initial viable cell number. (**b**) Proteome profiling of parental and short-term vemurafenib-treated persisting cells reflects the growth-arrested persistent state (downregulation of translation, metabolism and cell cycle proteins) and shows alterations in differentiation state markers (for example, upregulation of NGFR/CD271 for the persistent populations). Two parental melanoma cell lines (M229P and M238P) left untreated or were treated with 1 μm vemurafenib (vem) for 6 days (6d), and the corresponding long-term resistant cells (M229R and M238R), were profiled by quantitative mass spectrometry for proteome expression differences. For the heatmap representation the data was filtered for an analysis of variance *P*-value <0.05 and an average 3-fold up- or downregulation between the persistent and parental cell lines. Representative proteins in gene ontology groups discussed in the manuscript are indicated. (**c**, **d**) Flow cytometry confirms upregulation of NGFR/CD271 in the short-term persistent state for M229 (**c**) and M238 (**d**) cells. (**e**) Profiling of phospho-signaling alterations in the persistent cell populations. Three parental melanoma cell lines (M229P, M238P and M249P) were left untreated or were treated with 1 μm vemurafenib for 6 days (+vem(6d)) and phospho-proteome alterations were profiled by quantitative mass spectrometry. Data were filtered for a significant phosphorylation difference in the parental and short-term persistent populations (BH-corrected, Student’s *t*-test-based false discovery rate <0.2; and 2-fold up- or downregulation).

Having documented that long-term adaptation to vemurafenib treatment (that is, full resistance formation) engages a complex differentiation program, we asked how this long-term adaptive state compares with the short-term adaptive state of vemurafenib-treated melanoma cells. We and others have observed that melanoma cell lines treated with vemurafenib show only limited cell death concomitant with persistence of a slow-cycling subpopulation (Figure 4a, Supplementary Figure S5; [12, 17, 18, 35, 51]). This persistent cell subpopulation is thought to constitute a reservoir for the development of subsequent proliferative resistance [12, 18, 51].

Whole-proteome profiling and clustering demonstrated that the persisting cells are themselves in a distinct cellular state compared with both the parental, drug-naive cells and the chronically drug-exposed, proliferative resistant cells (Figure 4b, Supplementary Figures S6 and S7, Supplementary Table S5). Not surprisingly, the persistent state was clearly marked by the growth arrest-coherent downregulation of several translation-, metabolism- and cell cycle-related proteins. In addition, the persistent state was characterized by elevated expression of several melanoma de-differentiation marker proteins such as NGFR/CD271, ALCAM/CD166 and S100B: NGFR has been identified as a presumptive marker for melanoma-initiating/tumor stem cells [52], a neural-crest cell marker for desmoplastic and neurotropic melanomas [53] and a marker for immunotherapy-resistant, de-differentiated melanoma cells [54]; ALCAM was identified as a melanocytic stem cell marker, which shows higher expression for melanomas than banal nevi [55, 56]; and S100B has been associated with disease stage, reduced survival, metastases and treatment response [57]. Validation experiments for cell surface expression of NGFR/CD271 by flow cytometry demonstrated high upregulation in persisting cells, but even downregulation for one of the resistant cell lines (M238R; Figure 4c and d). In reverse, markers for the resistant state (PDGFRB and FN1) were not or were only slightly elevated in the persistent state (see below). In summary, although both long-term and short-term adaptation involve a differentiation state change, these states are distinct.

To gain insights into the signaling alterations occurring upon short-term BRAF inhibitor exposure and resulting in the persistent population—and to subsequently compare these with the fully resistant state—we conducted an additional global phospho-profiling experiment on three parental vs early drug-persistent pairs (from the aforementioned M229 and M238 parental lines as well as another BRAF inhibitor sensitive line M249; Figure 4e, Supplementary Figure S6, Supplementary Table S6). Persistence formation resulted in a distinct signaling state—with a higher consistency for the M229P and the M238P pairs than for the M249P pair (Supplementary Figure S8). Again, many downregulated phosphorylation sites, total proteins and the predicted downregulation of CDK1/2 signaling logically reflected the growth-downregulated state of the persistent cells, for example, downregulation of phosphorylated translation factors, metabolic enzymes and cell cycle proteins (Figure 4e, Supplementary Table S6). Several phosphorylation sites were consistently upregulated in the persistent cells, including PKC-alpha (PRKCA), NOTCH2, SP3 and Catenin Alpha-1 (CTNNA1).

### Although overall distinct, the early persisting subpopulation shares particular signaling alterations with the proliferative resistant state

**Figure 5:**
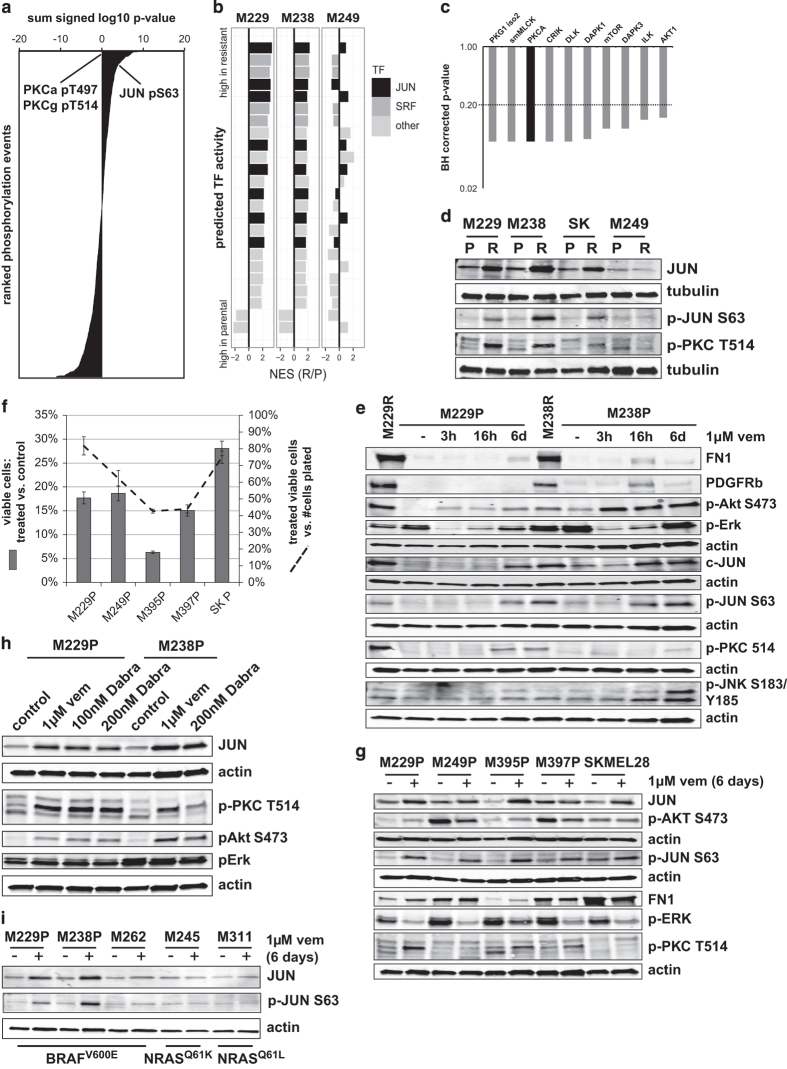
Shared signaling alterations of the short-term persistent (early adaption) and fully resistant (late adaption) cell populations include JUN upregulation and a PKC isoform switch. (**a**) Selection of shared phosphorylation events based on summed effect ranking. Phosphorylation sites were ranked by their summed signed log *t*-test values for the two resistant/parental and the two persistent/parental pairs (data collapsed on proteins by largest absolute sum). Among the top proteins, JUN and PKC were selected for follow-up investigations. Note that phospho-JUN upregulation was initially only detected for the resistant pairs, but evidence for its upregulation was obtained through a targeted analysis of the mass-spectrometry data set (Supplementary Figure S9). (**b**) Transcription factor (TF) activity analysis shows upregulation of JUN activity in receptor tyrosine kinase (RTK)-resistance mechanism cells. Genes were ranked by their fold change expression difference in parental vs resistant melanoma cells. Gene-set enrichment analysis (GSEA) was conducted to identify enriched transcription factor binding sites (MSigDB database c3). The results were filtered for binding sites specifically enriched for M229P/R5 and M238P/R1 (false discovery rate (FDR) <0.1), but not for M249P/R4 (FDR>0.2). Normalized enrichment scores (NES) for significance filtered TF binding sites are shown (sorted by the sum of the NES for M229 and M238). See Supplementary Table S8. (**c**) Kinase enrichment analysis points to upregulated kinase activities in RTK-resistance mechanism cells. These include Akt1, ILK and PKC-alpha (PKCA). Phospho-peptides were ranked by their mean fold change in resistant to parental cells and upregulated kinase activities were identified (filtered for a FDR (BH-adjusted *P*-value) <0.2). See Supplementary Table S2. (**d**) Western blot confirmation of (phospho-) JUN upregulation and the PKC isoform switch in RTK-resistance mechanism cells (M229R, M238R, SKMEL28R), but not in an NRAS-mutated resistant cell line (M249R). (**e**) Short-term vemurafenib-treated, persisting cells share alterations in JUN and protein kinase C (PKC) signaling with fully resistant melanoma cells. M229P and M238P were left untreated (−) or treated with 1μm vemurafenib/PLX4032 (vem) for 3 h, 16 h and 6 days and compared by western blot with the fully resistant cells (M229R and M238R). Short-term vemurafenib-treated cells share the upregulation of (phospho-) JUN and a PKC isoform switch with the fully resistant state. (**f**) An extended set of five parental melanoma cell lines demonstrates a persistent cell population after a 6- day treatment with 1 μm vemurafenib. The percent viable cells vs number of cells initially plated (dashed line) and the percent viable cells vs cells left untreated and cultured for 3 days (bars) is shown (*n*=3). (**g**) Western blot shows shared signaling alterations upon 6-day 1 μm vemurafenib treatment in these melanoma cell lines. (Phospho-) JUN upregulation is shared by all cell lines; the PKC isoform switch is most prominent for vemurafenib-treated M229P, M395P and M397P cells. (**h**) Vemurafenib (PLX) and dabrafenib (Dabra) treatment induce similar alterations of JUN and PKC signaling. Western blot of M238P and M229P melanoma cells treated for 6 days with vemurafenib or dabrafenib. (**i**) BRAF, but not NRAS-mutated melanoma cells show (phospho-) JUN upregulation upon 6-day vemurafenib (vem) treatment.

The short-term adaptation persistent cells are in an overall distinct cellular state with a different set of signaling alterations. However, we questioned whether long- and short-term adaptation to vemurafenib treatment share any signaling alterations that could potentially guide the identification of (co-) treatment approaches that prevent and/or target adaptive responses. To this end, we ranked the signaling alterations by a combined perturbation score incorporating the measured changes in the two fully resistant and two persistent cell line pairs (Figure 5a, Supplementary Table S7). The shared upregulated signaling events include PKC-alpha (PRKCA, pT497), JUN (pS63), progesterone receptor membrane component 1 (PGRMC1, pS57 and pS54), and tumor suppressor p53-binding protein 1 (TP53BP1, pS500 and pS380). Note that phospho-JUN upregulation was initially only detected in the resistant pairs, but clear evidence of its upregulation in the persistent cells was also obtained through a targeted analysis of the mass-spectrometry data set (Supplementary Figure S9) and by western blotting (Figure 5d)—for both the S63 and the S73 site of JUN (Supplementary Figure S10B). For both PKC-alpha and the transcription factor JUN, our data and analysis provided multiple lines of evidence implicating their role in the BRAF inhibitor adaptive states. In addition to its increase in net phosphorylation levels, JUN is transcriptionally upregulated in the resistant and the ‘invasive phenotype’ melanoma cells (Figure 3c). Moreover, a transcription factor binding site enrichment analysis of the RTK-resistance mechanism gene expression data predicted higher JUN activity for the resistant cells (Figure 5b and Supplementary Table S8). In the case of PKC-alpha, the kinase enrichment analysis of our phosphorylation data set implicated a higher PKC kinase activity (Figure 5c).

We further confirmed the Jun and PKC signaling alterations by western blot (Figure 5d). Upregulation of JUN and phospho-JUN is observed in an expanded panel of three resistant sublines with RTK-associated resistance but not in an alternate subline with mutant NRAS-driven resistance (M249R). Similarly, we observed alteration in the pattern of PKC isoform phosphorylation and upregulation of a single PKC isoform associated only with the RTK-resistance mechanism using the same set of cell line pairs. We confirmed that the resistance mechanism results specifically in upregulation of the PKC-alpha isoform using a knockdown strategy (Supplementary Figure S10A). We classified this phenomenon as a PKC isoform switch, as from three detectable isoforms only phosphorylation of PKC-alpha is strongly induced. Both the JUN and PKC signaling alterations are also detected in the early persistent cellular subpopulations (Figure 5e and Supplementary Figure S10B).

Next, we tested whether the cell cycle downregulation, as well as JUN and PKC signaling responses to short-term BRAF inhibitor treatment are common. We treated three additional BRAF^V600E^-positive melanoma cell lines with vemurafenib and found that each and every persistent cell population (Figure 5f) displayed consistent upregulation of JUN and phospho-JUN upon short-term BRAF inhibitor exposure. In contrast, the PKC isoform switch was observed in half of the lines (Figure 5g). To validate that these responses are on-target effects of vemurafenib, we repeated these experiments with dabrafenib, an alternative FDA-approved BRAF inhibitor ([7, 58]; Figure 5h). Both BRAF inhibitors show the same effects on both JUN upregulation and the PKC isoform switch. In addition, we find that the observed response is present for BRAF-mutated, but not NRAS-mutated melanoma cells, supporting these responses as on-target effects of the BRAF inhibitors (Figure 5i).

### Co-targeting mutant BRAF signaling and JUN function results in synergistic death and diminishes the persistent cell reservoir

With JUN upregulation and the PKC isoform switch as shared signaling alterations between the early persistent, survival and later, proliferative drug-resistant states, we next asked whether these alterations are optimal co-treatment targets. As initial experimentation showed that JUN knockdown, but not PKC-alpha knockdown, was effective in a vemurafenib co-treatment experiment, we focused our further efforts in this study on JUN (Supplementary Figure S11). Of note, in future studies, it will be pertinent to combine isoform-specific activity and substrate measurements to reveal the functional role of the PKC isoform switch.

**Figure 6:**
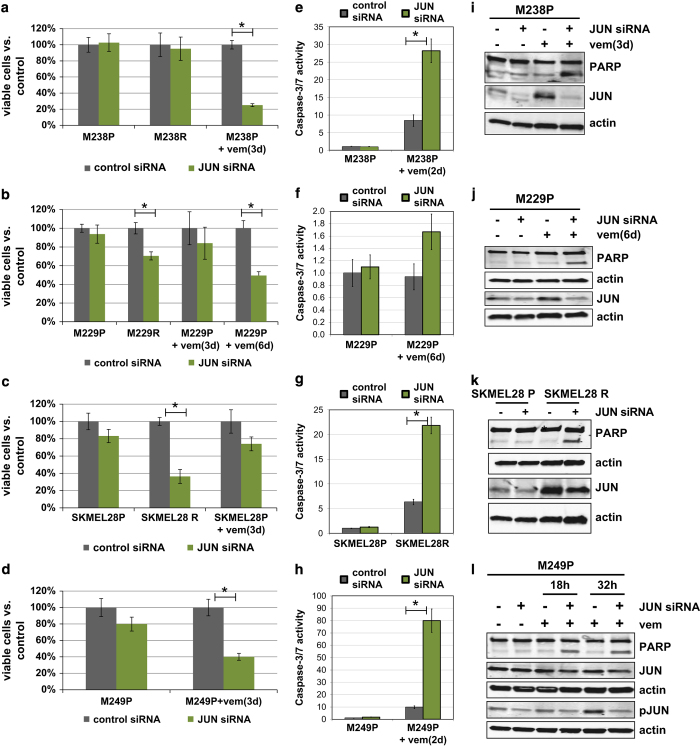
Co-targeting mutant BRAF signaling and JUN function results in synergistic death and diminishes the persistent cell reservoir. (**a**–**d**) Effect on cell viability of 1 μm vemurafenib and JUN short interfering RNA (siRNA) knockdown co-treatment for parental (P), fully resistant (R), up-front co-treated (+vem(3d)), and short-term persisting cell (+vem(6d)). Viable cells were counted by Trypan-blue staining with a ViCell counter. A JUN siRNA pool was used for the experiments in this figure, see Supplementary Table S12 for confirmation with individual siRNAs and Supplementary Table S13 for confirmation with distinct JUN short hairpin RNAs (shRNAs). (**e**–**h**) Induction of apoptosis (Caspase-3/7 activation) in melanoma cells co-treated with 1μm vemurafenib and JUN siRNA. (**i**–**l**) Western blot of melanoma cells co-treated with 1μm vemurafenib and JUN siRNA. PARP cleavage is shown as an apoptosis marker. Phospho-JUN was included for M249P cells owing to its lower upregulation of total JUN (see Figure 5g). Significant pairwise differences with a *t*-test *P*-value <0.05 are indicated (*). Overall, JUN siRNA knockdown had a significant effect on cell viability both for the panel of resistant (*P*-value=4E−6) and persisting cells (*P*-value<2E−16) and significantly induced apoptosis in the resistant/persisting cell line panel (*P*-value=1E−8), but not in the parental cell line panel (two-way analysis of variance *P*-values with JUN siRNA treatment and cell line as factors; significance level *α*=0.01).

JUN knockdown by itself only minimally affected untreated parental melanoma cells (Figure 6). In strong contrast, JUN knockdown induced substantial and significant death in our panel of both resistant cell lines and short-term vemurafenib-treated (that is, persistent) cultures (Figure 6a–d). Of note, the kinetics of JUN upregulation impacts the optimal sensitivity window for JUN knockdown: M229P cells show slower JUN upregulation (Figure 5e) and accordingly are most effectively co-targeted after longer vemurafenib pre-treatment (Figure 6b). Consistent with the death assays, in co-treated resistant and persistent cultures, we observe synergistic induction of apoptosis markers (Figure 6e–l). Note that we had selected the different timings for these experiments to accommodate for the different biological response times of the phenotypic effects on cell viability and apoptosis induction, as well as the response kinetics of the JUN knockdown procedure. Although there was a degree of expected cell line heterogeneity, overall, JUN siRNA knockdown had a significant effect on cell viability both for the panel of resistant (*P*-value=4E−6) and persisting cells (*P*-value <2E−16) and significantly induced apoptosis in the resistant/persisting cell line panel (*P*-value=1E−8), but not in the parental cell line panel. Finally, we corroborated the specificity for JUN in these responses with individual siRNAs (Supplementary Figure S12) and independent retroviral-based shRNA constructs (Supplementary Figure S13). With this, JUN upregulation represents a signaling alteration shared between the early adaptation (persistent population) and late adaptation (proliferative resistance) and that can be effectively up-front co-targeted to result in synergistic cell death and diminish the persistent cell reservoir.

### *In vivo* upregulation of JUN in short-term treated patients and disease progression melanoma biopsies

**Figure 7:**
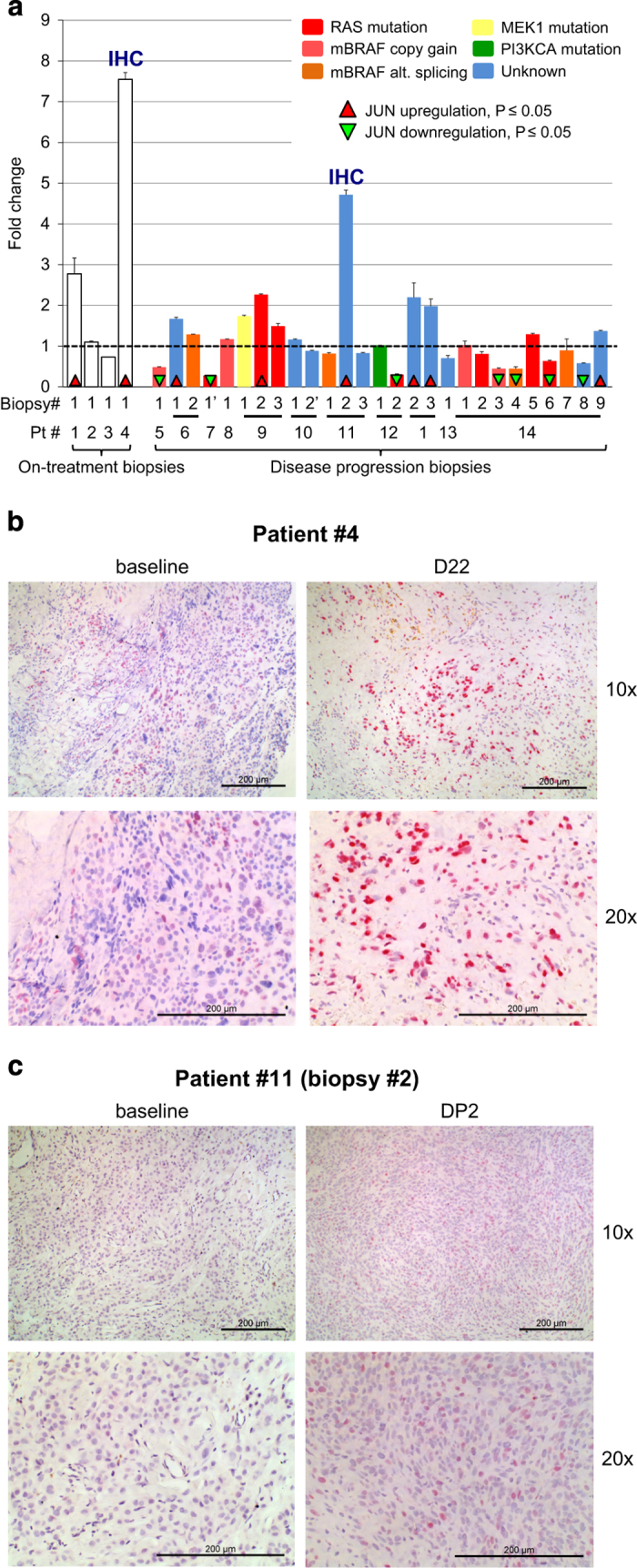
JUN upregulation is a common treatment response in melanoma patient samples (**a**) The occurrence of JUN upregulation on BRAFi treatment in mRNA expression data from 31 biopsies of 14 melanoma patients. The x-axis labels indicate patient (Pt) and biopsy numbers. Biopsy identification numbers with a quote symbol (‘) are from tumor(s) treated with BRAFi+MEKi dual inhibitor therapy. On the left are four on-treatment biopsies from four patients treated from 6 to 22 days, and on the right are disease progression (DP) biopsies annotated with their patient identification number (a total of 27 DP biopsies). The clinical details of the patients and the biopsied tumors are presented in Supplementary Table S9. mRNA expression was quantified by RNAseq except for Pt # 1, 13 and 14 for which exon-level microarray analysis was used. The JUN mRNA expression fold changes are computed with respect to each patient’s pre-treatment tumor JUN expression level. The JUN expression fold change for each patient is shown with error bars based on standard error of the mean (SEM) across replicates. Cases of statistically significant differential expression are indicated by up (red) and down (green) triangles. JUN was upregulated in two out of four on-treatment biopsies in two out of four patients and 6 out of 27 DP biopsies in 5 out of 11 patients. JUN was downregulated in 8 out of 27 biopsies in 4 out of 11 patients. DP biopsies from Pt #14 showed multiple cases of downregulation of JUN except for DP biopsy #9, indicating heterogeneous resistance mechanisms having a role [12]. Known genetic resistance mechanism identified in each sample are color coded as indicated in the key based on Shi *et al. *[12]. There is a slight enrichment of JUN upregulation in samples with an unknown resistance mechanism (Fisher’s exact test; *P*-value=0.095). (**b**, **c**) JUN immunohistochemistry staining of the on-treatment biopsy and the disease progression biopsy from **a** with substantial JUN mRNA upregulation. (**b**) patient #4; day 22 (D22) of kinase inhibitor treatment. (**c**) patient #11, biopsy #2, disease progression biopsy #2 (DP2). The respective baseline biopsies are also shown. JUN protein expression was visualized with a red chromogen. IHC, immunohistochemistry.

Finally, we evaluated JUN upregulation in patient samples. For this, we queried our patient-biopsy mRNA expression database containing on-treatment biopsies from four patients and 27 disease progression biopsies. On-treatment biopsies were obtained 6–22 days after treatment onset and the disease progression biopsies were obtained from patients on the progression of disease after the initial progression-free response (Supplementary Table S9). When compared with baseline JUN expression, two out of four on-treatment biopsies (two of four patients) and 6 out of 27 disease progression biopsies (5 of 11 patients) demonstrated significant upregulation of JUN mRNA levels (Figure 7a). There was a slight enrichment of JUN upregulation in disease progression samples that do not harbor an identified MAPK-pathway reactivation resistance mechanism. JUN protein-based immunohistochemistry staining of tumor sections confirmed the mRNA results (Figure 7b and c). With this, JUN upregulation is a common response to BRAF inhibitor treatment.

## Discussion

BRAF inhibitors including vemurafenib and dabrafenib, have provided unprecedented benefits for the treatment of metastatic melanoma [4, 6]. However, resistance development severely limits the rate and duration of clinical response to the BRAF inhibitors [59]. Although only a minority of patients carry tumors with pre-existing resistance and do not clinically respond to BRAF inhibitor treatment (intrinsic resistance), most patients show an initial response followed by progression with a median time of 6–7 months (acquired resistance). This is because BRAF inhibitor treatment can leave behind a persistent cell population that does not show full resistance (for example, shows a low proliferation rate) but serves as a cellular reservoir for the development of full-blown proliferative resistance [17, 18].

Several BRAF inhibitor resistance mechanisms have been identified. These are commonly classified into mechanisms that either reactivate the MAP-kinase pathway or mechanisms that activate alternative pathways such as the PI3K–AKT pathway. Among the former is the acquisition of secondary NRAS/KRAS mutations [9, 12] and the expression of a truncated BRAF variant [11, 60]; among the latter is the overexpression of PDGFRB [9] or IGF1R ([13]; RTK-associated resistance mechanisms). In addition, proteins that contribute to early persistence have been identified, including FOXD3 [61]. These previous studies have engendered clinical strategies such as BRAFi+MEKi or BRAFi+AKTi already being tested (and showing promise) in patients.

Here, we undertook an integrative proteomics/transcriptomic approach to understand the context and global alterations associated with resistance formation to devise novel BRAFi-based combinatorial strategies to suppress resistance. To this end, we have generated several quantitative protein, phospho-protein and ECM-protein expression data sets for different cellular states (parental, resistant, persistent). A challenge for the direct integration of the data sets (as seen for JUN phosphorylation, Supplementary Figure S9) was the often limited overlap of the detected proteins (Supplementary Figure S14). Pairwise comparisons demonstrate that although expected similarities exist, these data sets are consistent and largely complementary (Supplementary Figure S14). This is reflected in our integration approach presented here: rather than a one-shot data generation/integration step, each data set was generated by considering the previous insights and conceptually integrating them in a step-by-step manner to reveal the important factors in the mechanism of resistance.

At the onset of our study, the available data suggested that the RTK-resistance mechanism [9, 13, 62] takes place in a broader context of adaptive alterations. For example, pharmacological inhibition of PDGFRB by itself is not fully effective, and RTK-associated resistant cells show striking cellular morphological alterations [9]. In addition, in the current study, we did not find evidence for overall increased phospho-Tyr signaling in these cells (Supplementary Figures S1 and S2, Supplementary Table S1). Rather, consistent with other studies [34], we found that RTK-associated resistant cells express a mesenchymal gene signature (Figure 2), show extensive alterations in cytoskeleton and cell attachment-related signaling (Figure 1) and secrete ECM components that are associated with a mesenchymal state [43–45]. With this, the de-differentiation response of the resistant cells is reminiscent of EMT observed in other cancers [19, 21, 63]. EMT has been associated with metastasis formation, which is consistent with the observed cytoskeletal and invasive phenotype alterations. Moreover, EMT has been reported as a mechanism of resistance to kinase inhibitor therapies for several cancer types including lung, colorectal and breast cancer [25, 64, 65]. In these cases as well, the mesenchymal resistance phenotype is thought to involve a multitude of signaling alterations (for example, upregulation of several RTKs) rather than a single gene alteration or mutation [23, 64]. Importantly, PDGFRB upregulation is also a common component of these mesenchymal transition programs and its role in migration and resistance has been reported [66]. Taken together, PDGFRB and RTK family upregulation, the hallmark alteration of the analyzed resistance mechanism, is part of a more extensive EMT-like de-differentiation program that involves a multitude of signaling changes.

Differentiation state plasticity has also been reported before the BRAF inhibitor era—initially and most extensively in a series of reports by Hoek and Dummer [29–32]. In these reports, untreated (BRAF inhibitor-naive) melanoma cells were found to transition between a ‘proliferative’ and an ‘invasive’ state. In a direct gene signature comparison, we found a striking overlap between the resistant/de-differentiation state and the Hoek–Dummer ‘invasive’ state (Figure 3). The key feature of ‘invasive’ melanoma cells is their altered cell attachment and migration phenotype, phenotypes that we found to be also exhibited by BRAF inhibitor-resistant cells. In addition, published reports have demonstrated that MAPK-pathway inhibition in ‘proliferative’ melanoma cells can at least partially induce phenotypic features of the ‘invasive’ state [31]. Other examples for differentiation state plasticity in melanoma—with close ties to the resistant/invasive/mesenchymal state—include vasculogenic mimicry [67] and acquisition of stem-like properties [68]. Importantly, independent reports support the *in vivo* relevance of this mesenchymal de-differentiation state in BRAF inhibitor-naive melanoma. Bittner *et al.* [69] identified a related invasive phenotype cluster using short-term melanoma cultures. In addition, Lewis *et al.* [70] analyzed melanoma patient samples directly by RT-PCR and identified a cluster of samples that demonstrates features of the invasive/mesenchymal phenotype (for example, increased WNT5a, JUN, EGFR expression). Finally, in recent publications, we and others reported on the relevance of a closely related MITF^low^/AXL^high^ cellular state in melanoma resistance [36, 37]. With this, the observed differentiation state and phenotypic alteration of the BRAF inhibitor-resistant cells complements previous studies and clearly reflects the common cellular plasticity program of melanoma cells and tumors. Of note, the link established here between the de-differentiation and BRAF inhibitor resistance has also implications for other undesired outcomes such as the induction of an enhanced metastasis potential—for example, a recent retrospective analysis showed an increase in metastasis formation for patients that failed BRAF inhibitor treatment compared with dacarbazine treatment [71].

We and others have observed that vemurafenib treatment induces a slow-cycling, persistent cell population [17, 18, 35], which serves as a likely reservoir for the emergence of fully resistant proliferative cells [18]. On exploring the relationship between the early persistent and the fully resistant state using molecular profiling, we found the persistent state to be distinct from the fully resistant state and from the parental state. Specifically, clustering analysis separated the states (Supplementary Figure S7); markers for the resistant state (PDGFRB and FN1) were not or were only slightly elevated in the persistent state (Figure 5); and the persistent state showed distinct marker expression such as NGFR/CD271 (Figure 4). On the basis of its molecular program, the de-differentiation persistent state is potentially related to the NGFR/CD271^hi^ state reported by Landsberg *et al.* [54] as a resistance mechanism to adaptive T-cell therapy, and to the NGFR/CD271^hi^ state of melanoma stem cells [52, 72], which, however, is still debated [73].

Despite clear differences in the complex changes associated with the slow-cycling/persistent and resistant states, we asked whether these short-term and long-term adaptation programs share any potentially targetable signaling alterations. Among the identified shared signaling alterations between these states were JUN upregulation and a PKC isoform switch (Figure 5). In BRAF inhibitor-naive melanoma, JUN has been reported as a regulator of tumor progression and as a potential therapeutic target [74–77]. JUN upregulation has been associated with melanoma treatment resistance to classical chemotherapy [78]. In BRAF inhibitor-resistant melanoma cells, JUN was characterized as a mediator of upregulated PD-L1 expression [79]. Forced expression of the JUN binding partner FOS can confer resistance to RAF and MEK inhibitors [80]. In our study, JUN knockdown was synergistic with BRAF inhibition in co-treatment experiments, whereas JUN knockdown in parental cells had only a minimal effect, it prevented vemurafenib persistence and significantly decreased viability of resistant cells (Figure 6). In addition, we found that JUN upregulation is a common treatment response in melanoma patient samples (Figure 7). From this, we conclude that early and late vemurafenib non-responsive states share signaling alterations that can be effectively targeted *in vitro,* and we provide initial evidence that these alterations are observed *in vivo*.

Recently, in support of our finding, Fallahi-Sichani *et al.* [81] and Ramsdale *et al.* [82] have independently identified the relevance of JNK/c-JUN signaling in the adaptive response of melanoma cells to vemurafenib treatment. In our experiments, only JUN knockdown but not pharmacological JNK inhibition was effective (Figure 6, Supplementary Figure S15). A similar observation has been made previously by Spangler *et al.* [74]. In the study by Fallahi-Sichani *et al.,* a recently developed and improved JNK inhibitor JNK-IN-8 [83] was effective, whereas the JNK inhibitor SP600125 that was used in our study had a much more limited effect. Considering the strong inhibitory effect of SP600125 on JUN phosphorylation (Supplementary Figure S15) and the previously reported complexity of pharmacological JNK inhibition [84, 85], the implementation of JNK inhibition strategies in the clinic warrants a more detailed understanding of these differences in cellular response.

Despite the breakthrough that BRAF inhibitors brought to malignant melanoma therapy, major challenges lie ahead toward more effective and longer lasting treatments. The complexity of the adaptive response of melanoma cells—early in the form of persistence and later in the form of full resistance—poses a major challenge. Our results highlight the role of differentiation plasticity in the adaptive responses of melanoma cells. BRAF inhibitor-treated cells undergo changes far beyond single gene mutations and these differentiation changes are accompanied by altered dependencies and treatment sensitivity profiles. Notably, we identified shared signaling alterations between the early- and late-adaptive states and found that up-front co-targeting of BRAF and JUN results in synergistic killing and thus a marked reduction in the resistance-enabling persistent state. This result supports the therapeutic concept that up-front co-treatment regiments are necessary to overcome kinase inhibitor resistance and provide the next advance in genetically informed therapies [86, 87].

## Materials and Methods

An extended description of the methods is available in Supplementary Information.

### Cell culture

Melanoma cells were cultured in RPMI with 10% fetal bovine serum (FBS) and antibiotic-antimycotic (Invitrogen, Carlsbad, CA, USA). Resistant melanoma cells were cultured in the presence of 1μm vemurafenib/PLX4032 (ChemieTek, Indianapolis, IN, USA).

### Quantitative proteomics

Tyrosine phospho-profiles were analyzed using a quantitative mass-spectrometry method as described previously [7, 8]. Briefly, the cells were lysed by sonication in urea lysis buffer before quantitative analysis of affinity enriched phospho-tyrosine peptides by mass spectrometry. Relative amounts of the same phospho-peptide across sample runs were determined using custom software from our lab that conducts run–run alignments and integrates the area under the unfragmented (MS1) monoisotopic peptide peak [38, 39]. Global phospho-Tyr/Thr/Ser profiles were analyzed as described previously by quantitative mass spectrometry using a protocol that involves peptide fractionation by strong cation exchange chromatography and phospho-peptide enrichment with titania material [40]. To profile alteration of secreted ECM components, the melanoma cells were seeded on tissue culture dishes and kept at high density (full confluence) for 2 days. After a phosphate-buffered saline (PBS) wash, the cells were detached with 20 mm NH_4_OH and the plates were washed four times with PBS. The ECM components were detached and collected in ECM lysis buffer (8 m urea, 100 mm NH_4_HCO_3_ and 0.1 m DTT). The samples were processed for mass-spectrometry analysis using an adapted FASP procedure [88] and quantitative mass spectrometry was conducted as described for the phospho-profiling protocol. For whole-proteome expression profiling, the cells were washed with PBS, lysed (8 m urea, 50 mm Tris/HCl, pH=7.5) and the samples were processed for mass-spectrometry analysis using an adapted FASP procedure [88]. Peptides were analyzed by liquid chromatography tandem mass spectrometry as described for the phospho-profiling protocol and quantified with the Sieve software (version 2.0, Thermo Fisher Scientific, Waltham, MA, USA).

### Mesenchymal and phenotypic switch gene signatures

Expression data for the RTK-resistance mechanism (GSE24862; [9]) and the reference data sets (GSE4840, GSE4841, GSE4843, GSE22787, GSE20051; [30, 89, 90]) were obtained from the GEO repository and processed with R/Bioconductor [91]. Similarity to the ‘multi-cancer mesenchymal transition gene expression signature’ [47] was assessed with a permutation-based Kolmogorov–Smirnoff non-parametric rank test. The rank–rank-hypergeometric overlap method was used to evaluate the similarity with a mesenchymal signature derived from ‘a global map of human gene expression’ [50, 92].

### Attachment and migration phenotypic assays

For the sphere formation assay, wells of a 96-well plate were coated with 50 μl 1% (w/v) agarose in PBS. A total 15 000 cells were plated in 100 μl media per well and incubated overnight. The plate was shaken to distribute non-attached cells evenly before taking the images. For the detachment assay, 30 000 cells were plated per well of a 24-well plate and incubated for 2 days. The cells were PBS washed and trypsin-EDTA was added. After 3 min, RPMI media with 10% FBS was added and the detached cells were collected. The remaining (attached) cells were quickly washed with PBS and incubated with trypsin-EDTA until complete detachment. RPMI media with 10% FBS was added and the remaining cells were collected. The cells in both fractions were counted with a ViCell counter (Beckman Coulter, Indianapolis, IN, USA) and the percentage of cells detached after 3 min calculated. The tubuli-formation assay on Matrigel was performed similar to Zipser *et al.* [31]. Ninety-six-well plates were coated with 50 μl Matrigel (BD Bioscience, San Jose, CA, USA) per well. The cells were plated in 100 μl RPMI with 10% FBS (20 000 cells for M229P/R and 40 000 for M238P/R) and incubated overnight. The invasion assay was done similar to Zipser *et al.* [31]. Briefly, 24-well Transwell inserts (8-μm pore size, polycarbonate, Corning, Corning, NY) were coated with Matrigel (BD Biosciences). The cells were plated in serum-free media into the inserts, and media with 10% FBS were used as a chemoattractant. After 24 h, the cells were fixed with PFA and stained with crystal violet.

### Induction of persistent state by BRAF inhibition

Parental (BRAF inhibition sensitive) melanoma cells were plated at a density of 10 500 cells cm^−2^ in media without BRAF inhibitor. The next day, the media were exchanged and 1 μm vemurafenib (or the indicated concentration of dabrafenib) was added. The cells were cultured for 6–7 days with one media change after 3 days and another 1 day before analysis.

### Effects of JUN perturbations

Individual and pooled JUN siRNAs were obtained from Dharmacon/Thermo Scientific (Waltham, MA, USA) (siGENOME Human JUN (3725)). The siRNAs were reverse transfected with Lipofectamine RNAiMAX (Invitrogen). Lentiviral shRNAs for JUN were obtained from Sigma-Aldrich (St Louis, MO, USA) (TRCN0000010366, TRCN0000039590) and lentiviral particles were produced with a standard protocol (http://www.broadinstitute.org/rnai/public/resources/protocols). Trypan-blue staining and a ViCell counter was used for viable cell counting (Beckman Coulter). For MTS-based cell viability experiments, the CellTiter 96 AQueous One Solution Cell Proliferation Assay was used (Promega, Madison, WI, USA). Apoptosis induction was measured by a PARP cleavage western Blot and by an ApoTox-Glo assay for Caspase-3/7 activation (Promega).

### Analysis of patient samples

The mRNA expression of samples originating from patients (Pt) #1, 13 and 14 were analyzed using the Affymetrix exon-based microarray HuGene ST (Affymetrix, Santa Clara, CA, USA). The samples from patient #1 (three technical replicates per sample) were analyzed using HuGene ST version 1.0, whereas the samples from patients #13 and 14 (two technical replicates per sample) were analyzed using version 2.1. The fold changes of genes were computed based on log2 RMA-normalized expression values of the on-treatment samples or disease progression samples against their respective baseline (pre-treatment) samples. FDR-adjusted *P*-value of differential expression are computed using the limma R package. The mRNA expression of all the other patients were analyzed by RNAseq. Each sample was run on two separate lanes in the Illumina HiSeq 2000 sequencing platform (Illumina, San Diego, CA, USA) to generate two (technical) replicates per sample. The fold change of RNAseq samples were computed on the basis of the ratio of averaged RPKM values of the samples and their respective baselines. The FDR-adjusted *P*-value of the RPKM change are computed using the GFOLD program [93]. Immunohistochemistry was performed using c-Jun antibody #9165 from Cell Signaling Technology (Danvers, MA, USA) at a 1:400 dilution, MACH 2 Rabbit AP Polymer secondary antibodies (Biocare Medical, Concord, CA, USA, catalog #MALP525) and a red chromogen (Vulcan Fast Red—Biocare Medical, catalog #FR805H) with hematoxylin counterstaining.

## Notes

Supplementary Table S1

Supplementary Table S2A

Supplementary Table S2B

Supplementary Table S2C

Supplementary Table S2D

Supplementary Table S3A

Supplementary Table S3B

Supplementary Table S5

Supplementary Table S6A

Supplementary Table S6B
