# Supplementary material for: JUN dependency in distinct early and late BRAF inhibition adaptation states of melanoma
Source: Cell Discov. 2016 Sep 6;2:16028–. doi: 10.1038/celldisc.2016.28 (PMC5012007; doi:10.1038/celldisc.2016.28)
Supplement: Supplementary information [file celldisc201628-s1.pdf]

Extended Materials & Methods

JUN dependency in distinct early and late BRAF inhibition adaptation states of melanoma

Bjoern Titz[1,4], Anastasia Lomova[1,4], Allison Le[1,4], Willy Hugo[7], Xiangju Kong[7], Johanna ten Hoeve[1,4], Michael Friedman[1,4], Hubing Shi[7], Gatien Moriceau[7], Chunying Song[7], Aayoung Hong[7], Mohammad Atefi[6], Richard Li[1,4], Evangelia Komisopoulou[1,4], Antoni Ribas[1,2,3,6], Roger S Lo[1,3,7], and Thomas G Graeber[1,3,4,5]

[1] Department of Molecular and Medical Pharmacology, UCLA.

[2] Department of Surgery, Division of Surgical-Oncology, UCLA.

[3] Jonsson Comprehensive Cancer Center at UCLA.

[4] Crump Institute for Molecular Imaging, UCLA.

[5] California NanoSystems Institute, UCLA.

[6] Division of Hematology-Oncology, Department of Medicine, UCLA.

[7] Division of Dermatology, Department of Medicine, University of California Los Angeles, Los Angeles, California (UCLA).

General reagents and techniques

M229, M238, M249, M262, M245, M311, M395, and M397 melanoma cell lines were derived from patient biopsies in our research program at UCLA (UCLA IRB approval #11-003254), and the inhibitor resistant sublines were derived as described (10). Melanoma cells were cultured in RPMI with 10% FBS and antibiotic-antimycotic (Invitrogen, Carlsbad, CA). Resistant melanoma cells were cultured in the presence of 1uM vemurafenib/PLX4032 (ChemieTek). SP600125 (JNK inhibitor) was obtained from EMD Millipore.

The following antibodies were used for detection by Western Blot: anti-JUN (H-79, Santa Cruz), anti-phospho-JUN S63 (54B3, Cell Signaling), anti-phospho-PKC gamma T514 (Cell Signaling), anti-phospho-AKT S473 (Cell Signaling), anti-phospho-Erk1/2 T202/Y204 (Cell Signaling), anti-PARP (Cell Signaling), anti-phospho-JNK S183/Y185 (Cell Signaling), anti-FN1 (BD Biosciences), anti-PDGRB (Cell Signaling), anti-EGFR (Cell Signaling), and anti-ACTA2.

CD271/NGFR surface expression was analyzed by flow cytometry. CD271 was stained with a PE mouse anti-human CD271 antibody according to standard procedures (BD Biosciences, #560927). PE mouse IgG1 Kappa was used as the isotype control (BD Biosciences, #551436). Flow cytometry was performed in the UCLA Jonsson Comprehensive Cancer Center (JCCC) and Center for AIDS Research Flow Cytometry Core Facility.

Phospho-Tyrosine profiling by mass-spectrometry

Tyrosine phospho-profiles for M229P, M229P treated with 1uM PLX4032, and M229R5 treated with 1uM PLX4032 were analyzed using a quantitative mass-spectrometry based method as described previously [7,8]. Cells were treated with 1uM PLX4032 for 6h, washed with PBS, and lysed by sonication in urea lysis buffer (8 M urea, 1 mM vanadate, 50 mM Tris/HCl, pH = 7.5). Cell lysates were cleared by

centrifugation and filtration and protein concentrations were determined using a Bradford assay. 26 mg total protein was used for each sample. The samples were adjusted to the same volume with urea lysis buffer and 10% (v/v) 1M sodium phosphate buffer (pH = 7.5) was added. Samples were reduced with 5 mM DTT for 1h at 37°C, alkylated with 25 mM iodoacetamide for 1h at RT, and the reaction was quenched with 10 mM DTT for 30min at RT. The samples were dialyzed against 2 M urea buffer (2 M urea, 50 mM Tris/HCl, pH = 8.0). Proteins were digested with trypsin twice: first with 350 mg Worthington trypsin (Worthington, Lakewood, NJ, USA) for 3h at 37°C, then with 350 mg sequencing grade trypsin overnight at 37°C (Promega, Madison, WI, USA). The digested lysates were cleared by filtration and then pH adjusted to 2-3 with 5% TFA. Peptides were reverse-phase extracted using HyperSep C18 columns (Thermo): wetting with 50% ACN, equilibration with 0.1% TFA, wash with 0.1% TFA, and elution with 30% ACN and 0.1% TFA. Eluates were lyophilized overnight to complete dryness. Peptides were resuspended in 1.5 ml resuspension buffer (100 mM Tris/HCl, pH = 8.0) and adjusted to a pH of 7.4. 240 ul washed anti-phosphotyrosine (4G10 antibody) agarose conjugate (Millipore) was added and incubated at 4°C on the rotator overnight. The beads were washed 1x with 700 ul binding buffer (50 mM Tris/HCl, pH = 7.4), 3x with 450 ul binding buffer, 2x with 450 ul wash buffer (25 mM NH4HCO3, pH 7.5), and 1x with 600 ul wash buffer. The supernatant was discarded and the enriched phospho-peptides eluted with 450 ul 0.1% TFA for 15min at 37°C. The eluate was concentrated with a SpeedVac (Thermo) and reverse-phase extracted with C18 ZipTips (Millipore) following the manufacturer's instructions.

Phosphorylated peptides were analyzed by liquid chromatography tandem mass spectrometry (LC-MS/MS) using an Eksigent autosampler coupled with a NanoLC-2D pump (Eksigent, Dublin, CA) and a LTQ-Orbitrap (Thermo Fisher Scientific, Waltham, MA). The samples were loaded onto an analytical column (10 cm x 75 µm i.d.) packed with 5 µm Integrafit Proteopep2 300 Å C18 (New Objective, Woburn, MA). Peptides were eluted into the mass spectrometer using a HPLC gradient of 5% to 40%

Buffer B in 45 min followed by a quick gradient of 40% to 90% Buffer B in 10 min, where Buffer A contains 0.1% formic acid in water and Buffer B contains 0.1% formic acid in acetonitrile. All HPLC solvents were Ultima Gold quality (Fisher Scientific). Mass spectra were collected in positive ion mode using data dependent MS/MS acquisition of the top 5 most abundant peptides. Each sample was analyzed twice (replicate runs) using one-half of the sample. MS/MS spectra were searched using SEQUEST (Thermo Fisher Scientific) algorithm against a database containing the combined human-mouse IPI protein database (downloaded December 2006 from ftp.ebi.ac.uk). Search parameters included carboxyamidomethylation of cysteine as modification. Dynamic modifications included phosphorylation on tyrosine and oxidation on methionine. Results derived from database searching were filtered using the following criteria: Xcorr > 1.0(+1), 1.5(+2), 2(+3), peptide probability < 0.001, and dCn > 0.01 Bioworks version 3.2 (Thermo). We estimated the false positive rate of sequence assignments at 1% based on a composite target-reversed decoy database search strategy (3). Peptide peaks sequenced in some samples but not others were located in the remaining samples by aligning the chromatogram elution profiles using a dynamic time warping algorithm (4). Relative amounts of the same phosphopeptide across samples run together were determined using custom software from our lab to integrate the area under the unfragmented (MS1) monoisotopic peptide peak (1,2). Differentially phosphorylated peptides in M229P and M229R5 cells were identified with a false-discovery rate of 10% by calculating Benjamini-Hochberg (BH) adjusted t-test-derived p-values.

Phospho-Serine/Threonine/Tyrosine profiling by mass-spectrometry

Global phospho-profiles were analyzed for two isogenic parental/resistant melanoma cell line pairs (M229P/R5 and M238P/R1): parental cells not treated and treated with 1uM vemurafenib for 6h; resistant cells kept with 1uM vemurafenib. In addition, phospho-profiles for three parental/persistent cell line pairs (M229P, M238P, and M249P) were generated: the persistent state was induced as

described below and the media for both the parental and persistent cells was exchanged one day before lysis.

Phospho-peptides were enriched, identified, and quantified as described previously (5). Cells were washed with PBS and lysed by sonication in urea lysis buffer (8 M urea, 1 mM vanadate, 10 mM NaF, 10 mM β-glycerophosphate, 10 mM sodium pyrophosphate, and 50 mM Tris/HCl, pH = 7.5). Cell lysates were cleared by centrifugation and/or filtration and protein concentrations were determined using a Bradford assay. 24 mg total protein per sample was used for the parental/resistant set and 2.5 mg per sample for the parental/persistent set. Samples were adjusted to the same volume with urea lysis buffer. Samples were reduced with 5 mM DTT for 1h at 37°C, alkylated with 25 mM iodoacetamide for 1h at RT, and the reaction was quenched with 10 mM DTT for 30min at RT. The samples were either dialyzed (parental/resistant set) or diluted (parental/persistent set) to adjust the buffer to 2 M urea and pH = 8.0. Proteins were digested with 250 ug (parental/resistant set) or 25 ug (parental/persistent set) sequencing grade trypsin overnight at 37°C (Promega, Madison, WI, USA). The digested lysates were cleared by filtration (parental/resistant set) or centrifugation (parental/persistent set). The pH was adjusted to 2-3 with 5% TFA and peptides were reverse-phase extracted (HyperSep C18 columns (Thermo) for the parental/resistant set; SepPak Vac tc18 1cc cartridges (Waters) for the parental/persistent set). Eluates (40% ACN, 0.1% TFA) were lyophilized overnight to complete dryness. The samples were fractionated into three fractions by strong cation exchange (SCX) chromatography. For this, the samples were resuspended in SCX basis buffer (5 mM KH_2PO_4 (pH=2.65), 30% ACN, 5 mM KCl) and loaded onto a conditioned PolySULFOETHYL A (PolyLC) column (conditioning with ACN and SCX basis buffer). The flow-through was saved and combined with a buffer A wash to yield fraction "LW". Fraction "F1" was eluted with 17.5 mM KCl in SCX basis buffer, and fraction "F2" with 70 mM KCl in SCX basis buffer. ACN was evaporated (SpeedVac), the peptides were reverse-phase extracted (HyperSep C18 columns (Thermo) for the parental/resistant set; Empore C18 cartridges (3M) for the

parental/persistent set) and eluted with 50% ACN, 0.1% TFA. Titania material was conditioned with ACN, 0.2 M sodium phosphate, and equilibration buffer (45% ACN, 0.1% TFA, 150 mg/ml lactic acid). The peptide samples were added to the titania material, lactic acid was added to a final concentration of 150 mg/ml, and incubated for 45 min at RT. The titania material was washed three times with equilibration buffer and twice with rinsing buffer (45% ACN, 0.1% TFA). The (phospho-) peptides were eluted with 3% NH_3/H_2O, dried in a SpeedVac, and reverse-phase extracted with ZipTips (EMD/Millipore). The enriched phospho-peptides were analyzed by quantitative mass-spectrometry as for the "Phospho-Tyrosine profiling by mass-spectrometry" method. For the parental/resistant set, Sequest was run within the Bioworks framework as described. The ascore algorithm was used for more confident phosphorylation site assignments (3). For the parental/persistent set, Proteome Discoverer (version 1.3, Thermo) was used for the peptide search and significance filtering. The data was searched with Sequest against a human reference database with all isoforms from the UniProt database (version 01/2012). The search parameters included: 2 maximum missed trypsin cleavage sites, 15 ppm precursor mass tolerance, 0.8 Da fragment mass tolerance, carbamidomethyl-Cys as a static modification, Ser/Thr/Tyr phosphorylation and oxidation of Met as dynamic modifications. Significance scoring of identified peptides was done with the Percolator node of Proteome Discoverer, which uses a support vector machine model trained on actual and decoy search results (6). We filtered the dataset for a peptide FDR < 0.01. The PhosphoRS node was used for more confident phosphorylation site assignments. Peptide peaks sequenced in some samples but not others were located in the remaining samples by aligning the chromatogram elution profiles using a dynamic time warping algorithm (4). Relative amounts of the same phosphopeptide across samples run together were determined using custom software from our lab to integrate the area under the unfragmented (MS1) monoisotopic peptide peak (1,2).

For analysis of the parental/resistant set, the raw quantification values for the M229P/R5 samples were log normalized to correct for unequal sample loading. T-test p-values comparing parental and resistant

cells were calculated for each phospho-peptide ion separately for each fraction (LW, F1, and F2). P-values were multiple hypotheses testing adjusted using the Benjamini-Hochberg procedure (7) as implemented in the multtest package of R (8). Multiple detections of the same phosphorylation site (different charge states, fractions) were collapsed to the detection with the lowest BH-adjusted p-value (q-value). This q-value and fold-change [resistant vs. parental] was kept for each non-redundant phosphorylation site. In addition, the sum of the quantification values over all charge states and fractions was calculated and used for data presentation. For the parental/resistant set, the data was filtered for phosphorylation sites significantly altered in both cell line pairs: q-value < 0.2 and abs(log2(fold-change [resistant/parental]) > 1 for both cell line pairs.

For the parental/persistent set the phosphorylation site positional isomers, ie. different phosphorylation site assignments for the same phospho-peptide, were filtered: multiple positional isomers for the same spectrum were collapsed to the one(s) with the highest assignment probability (PhosphoRS probability), for each positional assignment (for each charge state) the assignment with the lowest PEP score was selected, and positional isomers with a PhosphoRS probability < 20% were discarded, if at least one positional isomer with higher assignment probability was identified. The data was quantile normalized for each sample and each fraction. T-test p-values comparing parental and persistent cells were calculated for each phospho-peptide ion and each fraction (LW, F1, and F2). P-values were multiple hypotheses testing adjusted using the Benjamini-Hochberg procedure (7) as implemented in the multtest package of R (8). Multiple detections of the same phosphorylation site (different charge states, fractions) were collapsed to the detection with the lowest BH-adjusted p-value (q-value). This q-value and fold-change [persistent vs. parental] was kept for each non-redundant phosphorylation site. In addition, the sum of the quantification values over all charge states and fractions was calculated and used for data presentation.

Proteome profiling of extracellular matrix components

To profile alteration of secreted extracellular matrix (ECM) components the melanoma cells were seeded on tissue culture dishes and kept at high density (full confluence) for two days. After a PBS wash the cells were detached with 20mM NH_4OH and the plates were washed four times with PBS. The ECM components were detached and collected in ECM lysis buffer (8M urea, 100mM NH_4HCO_3, and 0.1M DTT). The samples were processed for mass-spectrometry analysis using an adapted FASP procedure (9). For this, the samples were transferred into an Amicon Ultra-0.5ml (10K NMWL) centrifugal filter and concentrated (centrifugation at 14,000xg for 20min). 200ul buffer UA (8M urea, 100mM Tris/HCl, pH=8.0) was added and concentrated (centrifugation at 14,000xg for 20min). This was repeated once. 100ul 50mM iodoacetamide in UA was added and the samples incubated for 10min in the dark. The samples were concentrated (centrifugation at 14,000xg for 20min) and washed thrice with 100ul buffer UB (2M urea, 100mM Tris/HCl, pH=8.0; centrifugation at 14,000xg for 20min). The filter was transferred to a new collection tube and 150ul digestion solution (2.5ug sequencing-grade trypsin [Promega], 1mM CaCl2, 1500U PNGase F [NEB, glycerol-free] in UB buffer) was added for an overnight incubation at 37C in a wet chamber. Peptides were eluted by centrifugation at 14,000xg for 20min and three additions of 50ul 0.5M NaCl and subsequent centrifugation at 14,000xg for 20min. Samples were acidified with TFA and desalted with a C18 desalting protocol.

Peptides were analyzed by liquid chromatography tandem mass spectrometry as for the phospho-profiling protocol. Proteome Discoverer (version 1.3, Thermo) was used for the peptide search and significance filtering. The data was search with Sequest against a human reference database with all isoforms from the UniProt database (version 01/2012). The search parameters included: 2 maximum missed trypsin cleavage sites, 15 ppm precursor mass tolerance, 0.8 Da fragment mass tolerance, carbamidomethyl-Cys as a static modification, and oxidation of Met and Pro and deamidation of Asn

(PNGase F products) as dynamic modifications. Significance scoring of identified peptides was done with the Percolator node of Proteome Discoverer, which uses a support vector machine model trained on actual and decoy search results (6). We filtered the dataset for a peptide FDR < 1% and at least two peptides per protein. In addition, the identified proteins were filtered for evidence of an extracellular localization (from UniProt database and GO terms).

Peptides were quantified by MS1 elution peak integration as described for the phospho-profiling protocols. The quantification values for each peptide were scaled by their mean values and protein quantification values were calculated as the median of the peptide quantification values. Proteins with significantly altered expression were selected controlling for a false-discovery rate of 0.2 (Student's t-test, Benjamini-Hochberg multiple hypothesis testing correction).

Whole proteome expression profiling

After a PBS wash the cells were resuspended in lysis buffer (8M urea, 50mM Tris/HCl, pH=7.5) and sonicated. The samples were clarified by centrifugation (10min at maximum speed) and protein concentrations were determined by a Bradford assay. The samples were processed for mass-spectrometry analysis using an adapted FASP procedure (9). For each sample 50ug protein was transferred into an Amicon Ultra-0.5ml (10K NMWL) centrifugal filter. DTT was added to a final concentration of 0.1M and incubated for 30min at 37C. The samples were concentrated (centrifugation at 14,000xg for 20min). 200ul buffer UA (8M urea, 100mM Tris/HCl, pH=8.0) was added and concentrated (centrifugation at 14,000xg for 20min). This was repeated once. 100ul 50mM iodoacetamide in UA was added and the samples incubated for 10min in the dark. The samples were concentrated (centrifugation at 14,000xg for 20min) and washed thrice with 150ul buffer UB (2M urea, 100mM Tris/HCl, pH=8.0; centrifugation at 14,000xg for 20min). The filter was transferred to a new collection tube and 150ul digestion solution (2ug sequencing-grade trypsin [Promega] and 1mM $CaCl_2$ in

UB buffer) was added for overnight incubation at 37C in a wet chamber. Peptides were eluted by centrifugation at 14,000xg for 20min and three additions of 50ul 0.5M NaCl and subsequent centrifugation at 14,000xg for 20min. Samples were acidified with TFA and desalted with a C18 desalting protocol.

Peptides were analyzed by liquid chromatography tandem mass spectrometry as for the phospho-profiling protocol. Proteome Discoverer (version 1.3, Thermo) was used for the peptide search and significance filtering. The data was search with Sequest against a human reference database with all isoforms from the UniProt database (version 01/2012). The search parameters included: 2 maximum missed trypsin cleavage sites, 15 ppm precursor mass tolerance, 0.8 Da fragment mass tolerance, carbamidomethyl-Cys as a static modification, and oxidation of Met and Pro as dynamic modifications. Significance scoring of identified peptides was done with the Percolator node of Proteome Discoverer, which uses a support vector machine model trained on actual and decoy search results (6). We filtered the dataset for a peptide FDR < 0.01.

Quantification was done with Sieve (version 2.0, Thermo) using standard parameters and a maximum frame number of 10,000. Frame-level data was quantile normalized by sample and mean scaled by frame. The frame-level data was collapsed on genes/proteins by median. The data was filtered for an ANOVA p-value < 0.05 (for the parental, resistant, and persistent groups) and three-fold up- or down-regulation comparing the persistent and parental group for the heatmap representation.

Mesenchymal and phenotypic switch gene signatures

The expression data for RTK-resistance mechanism cells were obtained from the GEO repository (GSE24862) (10). The data was RMA normalized and annotated using the R/Bioconductor package and the affy, genefilter, and annotate libraries (11). Processed data sets from Hoek et al. (GSE4840, GSE4841, GSE4843; GPL96 only) (12), Bloethner et al. (GSE22787) (13), and Joseph et al. (GSE20051) (14)

data sets were obtained from the GEO repository. The log2 fold-change values between classes were calculated and multiple probes for each gene were collapsed to the largest absolute log2 fold-change value before calculation of the pair-wise Pearson correlation coefficients. The correlation matrix was clustered with a hierarchical, Euclidean distance-based complete or single linkage clustering procedure.

For comparison of RTK-resistance mechanism signatures with the "multi-cancer mesenchymal transition gene expression signature"(15), we tested for significant enrichment of mesenchymal signature genes at the top of gene lists ranked by over-expression in resistant cells compared to their isogenic, sensitive counterparts. For this, expression data for the M229P/R, M238P/R, and M249P/R pairs from Nazarian et al. (10) were used. Genes were ranked by their fold-change up-regulation in resistant cells, with individual gene probes collapsed to the largest absolute fold-change and statistical significance was assessed with a permutation based Kolmogorov-Smirnoff (KS) non-parametric rank test (1000 permutations).

For comparison of the RTK-resistance mechanism signature with the mesenchymal signature derived from "a global map of human gene expression" (16), we obtained the "global" expression data from ArrayExpress (http://www.ebi.ac.uk/gxa/array/U133A). We defined samples annotated with "mesenchymal stem cell" as a class and calculated t-scores comparing this class with all other samples. Individual probes for each gene were collapsed to the probe with the largest absolute t-score. The rank-rank-hypergeometric (RRHO) overlap (17) between this signature and the sum fold-change signature for M229R/P and M238R/P (RTK-upregulated resistance mechanism) was calculated with the online RRHO tool (http://systems.crump.ucla.edu/rankrank/).

Kinase enrichment analysis

Identified phosphorylation sites were annotated with potential upstream kinases from the PhosphoSitePlus® database (18). For this, the provided kinase-substrate database was downloaded

(June 2013) and non-human phosphorylation sites were mapped to the orthologous phosphorylation sites of human proteins (alignment of “best-best” blast hits with needle (19)). Detected phosphorylation sites were annotated with these potential upstream kinases (Table E2 & 6). A GSEA-motivated statistic was used to identify significantly up- or down-regulated kinase activities (20). Phosphorylation sites were ranked by their fold-change up- or down-regulation in resistant or persistent melanoma cells. Phosphorylation site peptide variants (e.g., different charge states) were collapsed to the variant with the absolute largest $\log_2$ fold-change. Enrichment of phosphorylation sites linked to a specific upstream kinase were scored with a weighted Kolmogorov–Smirnov statistic (ks-score). Permutation-based p-values were calculated by comparing the actual ks-score with the ks-scores calculated for 1000 random permutations of the kinase assignments. These p-values were controlled for a false-discovery rate of 20% using the Benjamini-Hochberg procedure (7).

Attachment and migration phenotypic assays

For the sphere formation assay, wells of a 96-well plate were coated with 50ul 1% (w/v) agarose in PBS. 15,000 cells were plated in 100ul media per well and incubated overnight. The plate was shaken to distribute non-attached cells evenly before taking the images.

For the detachment assay, 30,000 cells were plated per well of a 24-well plate and incubated for two days. Cells were PBS washed and trypsin-EDTA was added. After 3min RPMI media with 10%FBS was added and the detached cells were collected. The remaining (attached) cells were quickly PBS washed and incubated with trypsin-EDTA until complete detachment. RPMI media with 10%FBS was added and the remaining cells were collected. Cells in both fractions were counted with a ViCell counter and the percentage of cells detached after 3min calculated.

The tubuli-formation assay on Matrigel was performed similar to Zipser et al. (21). 96-well plates were coated with 50ul Matrigel (BD Bioscience) per well. Cells were plated in 100ul RPMI with 10% FBS (20,000 cells for M229P/R and 40,000 for M238P/R) and incubated overnight.

The invasion assay was done similar to Zipser et al. (21). Briefly, 24-well Transwell inserts (8-um pore size, polycarbonate, Corning) were coated with Matrigel (BD Biosciences). Cells were plated in serum-free media into the inserts and media with 10% FBS was used as a chemoattractant. After 24h, the cells were fixed with PFA and stained with crystal violet.

Induction of persistent state by BRAF-inhibition

We and others have observed that BRAF-inhibition induces a growth-arrested, persistent state in melanoma cells (22,23). Parental (BRAF-inhibition sensitive) melanoma cells were plated at a density of 10.500 cells / cm^2 in media without BRAF inhibitor. The next day the media was exchanged and 1uM vemurafenib (or the indicated concentration of dabrafenib) was added. The cells were cultured for 6-7 days with two media changes (after 3 days, and then one day before analysis) and analyzed.

Effects of JUN perturbations

Individual and pooled JUN siRNAs were obtained from Dharmacon/Thermo (siGENOME Human JUN (3725)). siRNAs were reverse transfected with Lipofectamine RNAiMAX (Invitrogen, Carlsbad, CA). Lentiviral shRNAs for JUN were obtained from Sigma Aldrich (TRCN0000010366, TRCN0000039590) and lentiviral particles were produced with a standard protocol (http://www.broadinstitute.org/rnai/public/resources/protocols). Trypan-blue staining and a ViCell counter was used for viable cell counting (Beckman Coulter). For MTS-based cell viability experiments the CellTiter 96® AQueous One Solution Cell Proliferation Assay was used (Promega). Apoptosis

induction was measured by PARP cleavage on a Western Blot and by an ApoTox-Glo assay for Caspase-3/7 activation (Promega).

Analysis of patient samples

mRNA expression of samples originating from patient (Pt) # 1, 13 and 14 were analyzed using the Affymetrix exon-based microarray HuGene ST. Samples from patient # 1 (3 technical replicates per sample) were analyzed using HuGene ST version 1.0 while samples from patient # 13 and 14 (2 technical replicates per sample) were analyzed using version 2.1. Raw CEL files were RMA normalized using the oligoR package. The fold change of Microarray are computed based on log2 RMA-normalized expression values of the on treatment samples or disease progression (DP) samples against their respective baseline (pre-treatment) samples. FDR adjusted P-value of differential expression are computed using the limma R package.

mRNA expression of all other patients were analyzed by RNA seq. Each sample was run on two separate lanes in the Illumina HiSeq 2000 sequencing platform to generate two (technical) replicates per sample. The fold change of RNAseq samples were computed based on the ratio of averaged RPKM values of the samples and their respective baselines. The FDR adjusted p-value of the RPKM change are computed using the GFOLD program (24).

Immunohistochemistry was performed using c-Jun antibody #9165 from Cell Signaling Technology at a 1:400 dilution, MACH 2 Rabbit AP Polymer secondary antibodies (Biocare Medical, cat. # MALP525) and a red chromogen (Vulcan Fast Red - Biocare Medical, cat. #FR805H) with hematoxylin counterstaining.

References

1. Rubbi L, Titz B, Brown L, Galvan E, Komisopoulou E, Chen SS, et al. Global phosphoproteomics reveals crosstalk between Bcr-Abl and negative feedback mechanisms controlling Src signaling. Sci Signal. 2011;4:ra18.

2. Skaggs BJ, Gorre ME, Ryvkin A, Burgess MR, Xie Y, Han Y, et al. Phosphorylation of the ATP-binding loop directs oncogenicity of drug-resistant BCR-ABL mutants. Proc Natl Acad Sci U S A. 2006;103:19466–71.

3. Beausoleil SA, Villén J, Gerber SA, Rush J, Gygi SP. A probability-based approach for high-throughput protein phosphorylation analysis and site localization. Nat Biotechnol. 2006;24:1285–92.

4. Prakash A, Mallick P, Whiteaker J, Zhang H, Paulovich A, Flory M, et al. Signal maps for mass spectrometry-based comparative proteomics. Mol Cell Proteomics MCP. 2006;5:423–32.

5. Zimman A, Chen SS, Komisopoulou E, Titz B, Martínez-Pinna R, Kafi A, et al. Activation of aortic endothelial cells by oxidized phospholipids: a phosphoproteomic analysis. J Proteome Res. 2010;9:2812–24.

6. Käll L, Canterbury JD, Weston J, Noble WS, MacCoss MJ. Semi-supervised learning for peptide identification from shotgun proteomics datasets. Nat Methods. Nature Publishing Group; 2007;4:923–5.

7. Benjamini Y, Hochberg Y. Controlling the false discovery rate: a practical and powerful approach to multiple testing. J R Stat Soc Ser B CN - 13800. 1995;289–300.

8. Pollard KS, Gilbert HN, Ge Y, Taylor S, Dudoit S. multtest: Resampling-based multiple hypothesis testing. 2008.

9. Wiśniewski J, Zougman A, Nagaraj N, Mann M. Universal sample preparation method for proteome analysis. Nat Methods. 2009;6:359–62.

10. Nazarian R, Shi H, Wang Q, Kong X, Koya RC, Lee H, et al. Melanomas acquire resistance to B-RAF(V600E) inhibition by RTK or N-RAS upregulation. Nature. 2010;468:973–7.

11. Gentleman RC, Carey VJ, Bates DM, Bolstad B, Dettling M, Dudoit S, et al. Bioconductor: open software development for computational biology and bioinformatics. Genome Biol. 2004;5.

12. Hoek KS, Schlegel NC, Brafford P, Sucker A, Ugurel S, Kumar R, et al. Metastatic potential of melanomas defined by specific gene expression profiles with no BRAF signature. Pigment Cell Res. 2006;19:290–302 LA – en.

13. Bloethner S, Hemminki K, Thirumaran RK, Chen B, Mueller-Berghaus J, Ugurel S, et al. Differences in global gene expression in melanoma cell lines with and without homozygous deletion of the CDKN2A locus genes. Melanoma Res. 2006;16:297–307.

14. Joseph EW, Pratilas CA, Poulikakos PI, Tadi M, Wang W, Taylor BS, et al. The RAF inhibitor PLX4032 inhibits ERK signaling and tumor cell proliferation in a V600E BRAF-selective manner. Proc Natl Acad Sci U S A. 2010;107:14903–8.

15. Cheng W-Y, Kandel JJ, Yamashiro DJ, Canoll P, Anastassiou D. A multi-cancer mesenchymal transition gene expression signature is associated with prolonged time to recurrence in glioblastoma. Harrison JK, editor. PLoS One. Public Library of Science; 2012;7:e34705.

16. Lukk M, Kapushesky M, Nikkilä J, Parkinson H, Goncalves A, Huber W, et al. A global map of human gene expression. Nat Biotechnol. Nature Publishing Group; 2010;28:322–4.

17. Plaisier SB, Taschereau R, Wong JA, Graeber TG. Rank-rank hypergeometric overlap: identification of statistically significant overlap between gene-expression signatures. Nucleic Acids Res. 2010;38.

18. Hornbeck P V, Kornhauser JM, Tkachev S, Zhang B, Skrzypek E, Murray B, et al. PhosphoSitePlus: a comprehensive resource for investigating the structure and function of experimentally determined post-translational modifications in man and mouse. Nucleic Acids Res. 2012;40:D261–70.

19. Rice P, Longden I, Bleasby A. EMBOSS: the European molecular biology open software suite. Trends Genet. 2000;16:276–7.

20. Subramanian A, Tamayo P, Mootha VK, Mukherjee S, Ebert BL, Gillette MA, et al. Gene set enrichment analysis: A knowledge-based approach for interpreting genome-wide expression profiles. Proc Natl Acad Sci U S A. 2005;102:15545–50.

21. Zipser MC, Eichhoff OM, Widmer DS, Schlegel NC, Schoenewolf NL, Stuart D, et al. A proliferative melanoma cell phenotype is responsive to RAF/MEK inhibition independent of BRAF mutation status. Pigment Cell Melanoma Res. 2011;24:326–33.

22. Abel E V, Aplin AE. FOXD3 is a mutant B-RAF-regulated inhibitor of G(1)-S progression in melanoma cells. Cancer Res. 2010;70:2891–900.

23. Basile KJ, Abel E V, Aplin AE. Adaptive upregulation of FOXD3 and resistance to PLX4032/4720-induced cell death in mutant B-RAF melanoma cells. Oncogene. 2011;

24. Feng J, Meyer CA, Wang Q, Liu JS, Shirley Liu X, Zhang Y. GFOLD: a generalized fold change for ranking differentially expressed genes from RNA-seq data. Bioinformatics. 2012;28:2782–8.
