# Supplementary material for: JUN dependency in distinct early and late BRAF inhibition adaptation states of melanoma
Source: Cell Discov. 2016 Sep 6;2:16028–. doi: 10.1038/celldisc.2016.28 (PMC5012007; doi:10.1038/celldisc.2016.28)
Supplement: Supplementary Figure S1 [file celldisc201628-s2.pdf]

Titz et al. Supplementary Figure 1

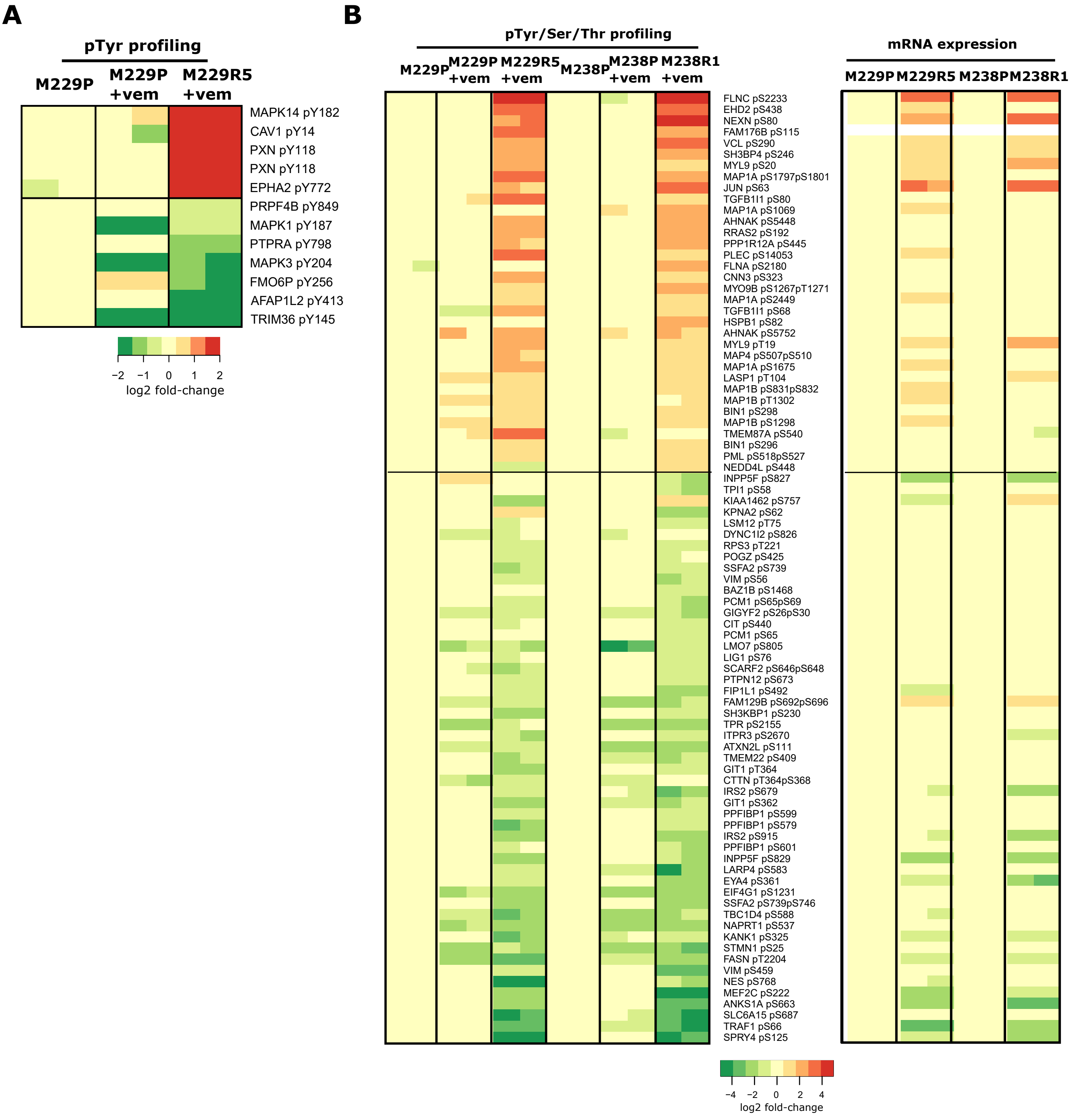

Phospho-profiling of vemurafenib resistant melanoma cells with RTK up-regulation. (A) Global phospho-Tyr profiling of M229P cells, M229P cells treated with 1uM vemurafenib/ PLX4032 (M229P+vem), and M229R5 cells treated with 1uM PLX4032 (M229R5+vem). Tyr-phosphorylated peptides were affinity enriched and quantified using a label-free mass-spectrometry procedure. Phospho-peptides differentially expressed in parental and resistant cells are shown in this peptide by sample matrix with the log2 fold-change represented by the color scale (FDR controlled at 0.1 level using Benjamini-Hochberg procedure). Phosphorylation of the focal adhesion components paxillin (PXN) and caveolin (CAV1) is highly up-regulated in the resistant cells. (B) Global (phospho-Tyr/Ser/Thr) signaling alterations and corresponding mRNA-expression differences in vemurafenib resistant cells with RTK up-regulation. Details of phospho-profiling results shown in Figure 1A and corresponding mRNA-expression data from Nazarian et al. (1).
