# Supplementary material for: JUN dependency in distinct early and late BRAF inhibition adaptation states of melanoma
Source: Cell Discov. 2016 Sep 6;2:16028–. doi: 10.1038/celldisc.2016.28 (PMC5012007; doi:10.1038/celldisc.2016.28)
Supplement: Supplementary Figure S2 [file celldisc201628-s3.pdf]

Titz et al. Supplementary Figure S2

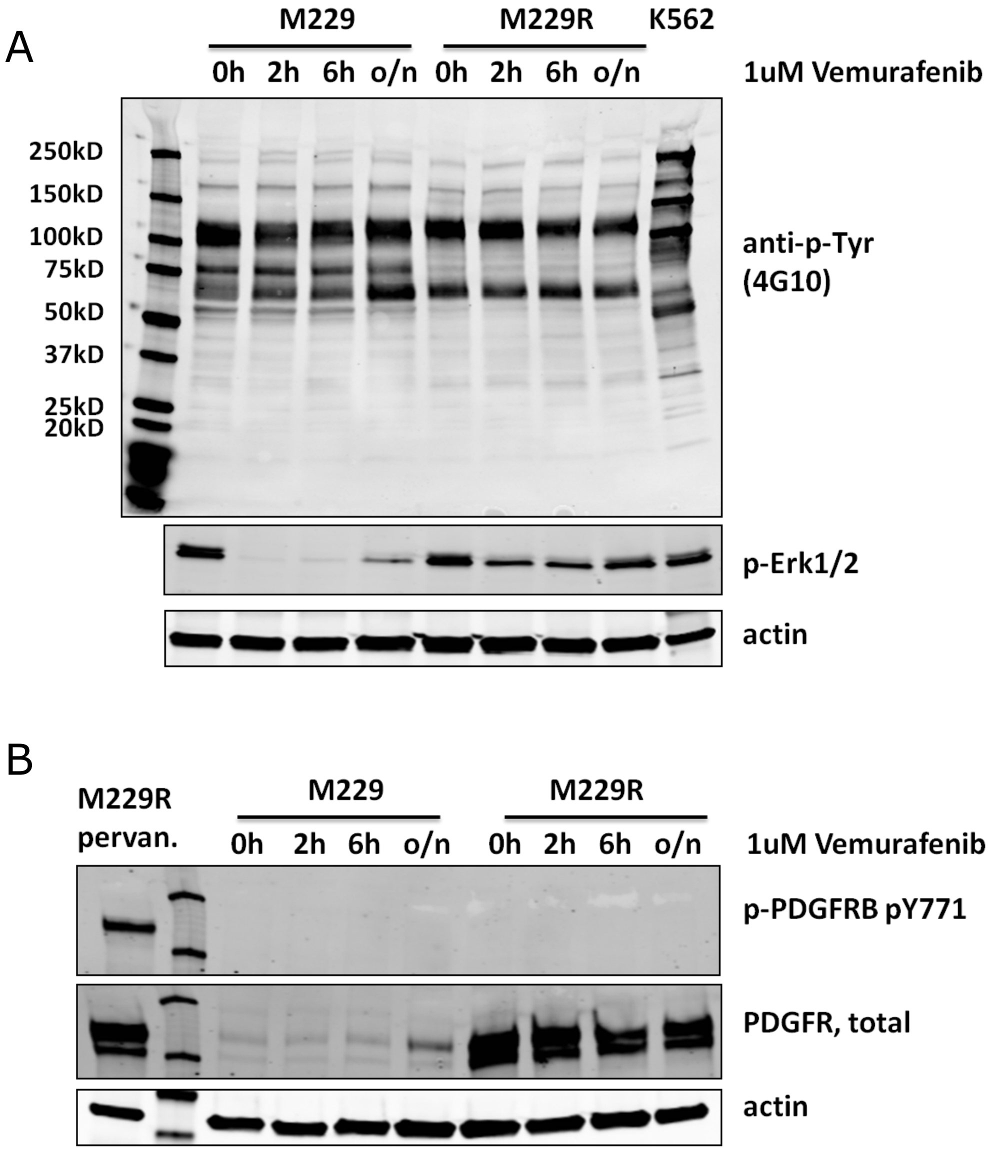

Western blots of phospho-Tyr signaling in M229 parental/resistant cells. (A) Parental M229 and resistant M229R5 (PDGFRB-/RTK-resistance mechanism) cells were treated with 1uM vemurafenib for 0h, 2h, 6h, and overnight (o/n) and their global phospho-Tyr signaling (4G10 antibody) and Erk phosphorylation was compared with the BCR-ABL+ cell line K562 as a control. Note that no sign of increased (global) phospho-Tyr signaling is visible in M229R cells. (B) Same experiment as in (A), but with pervanadate treated M229R cells as the control and probing for total and phospho-PDGFRB (Y771). Note the high up-regulation of total PDGFRB expression in M229R, but no sign of increased PDGFRB phosphorylation in M229R cells compared to the pervanadate treated control.
