# Supplementary material for: JUN dependency in distinct early and late BRAF inhibition adaptation states of melanoma
Source: Cell Discov. 2016 Sep 6;2:16028–. doi: 10.1038/celldisc.2016.28 (PMC5012007; doi:10.1038/celldisc.2016.28)
Supplement: Supplementary Figure S3 [file celldisc201628-s4.pdf]

Titz et al. Supplementary Figure S3

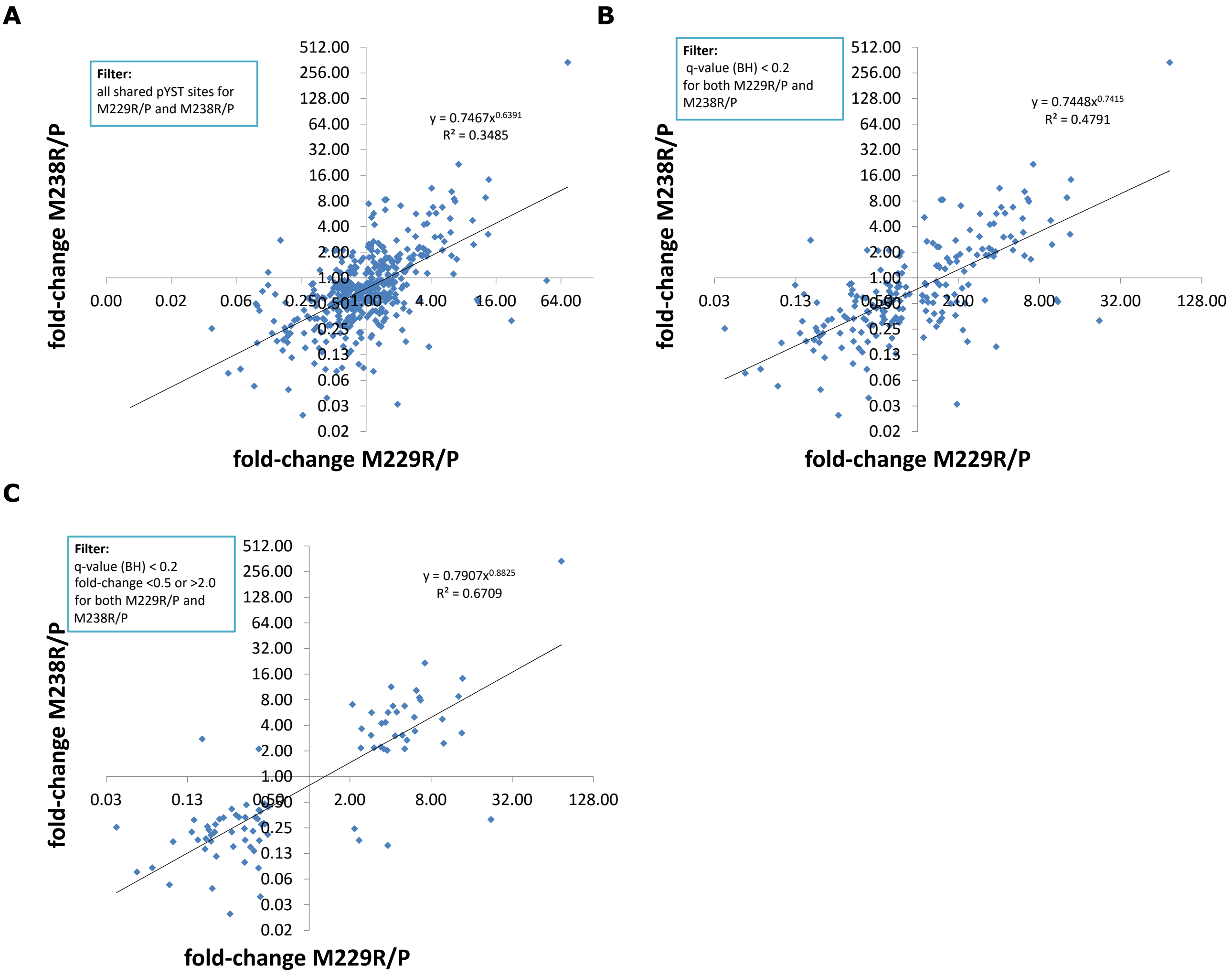

Correlation between phospho-profiling results for both isogenic pairs. Scatter plots comparing phospho-site fold-changes measured for M229 and M238 isogenic pairs (resistant vs. parental cell line). (A) All phospho-sites identified for both isogenic pairs. (B) Shared phospho-sites filtered for a q-value (FDR) < 0.2 for each isogenic pair. (C) As in B, but also filtered for a 2-fold up- or down-regulation for each isogenic pair. The fit to a power law is shown. The most stringent filter used in C was used for data representation and analysis in Figure 1.
