# Supplementary material for: JUN dependency in distinct early and late BRAF inhibition adaptation states of melanoma
Source: Cell Discov. 2016 Sep 6;2:16028–. doi: 10.1038/celldisc.2016.28 (PMC5012007; doi:10.1038/celldisc.2016.28)
Supplement: Supplementary Figure S4 [file celldisc201628-s5.pdf]

Titz et al. Supplementary Figure S4

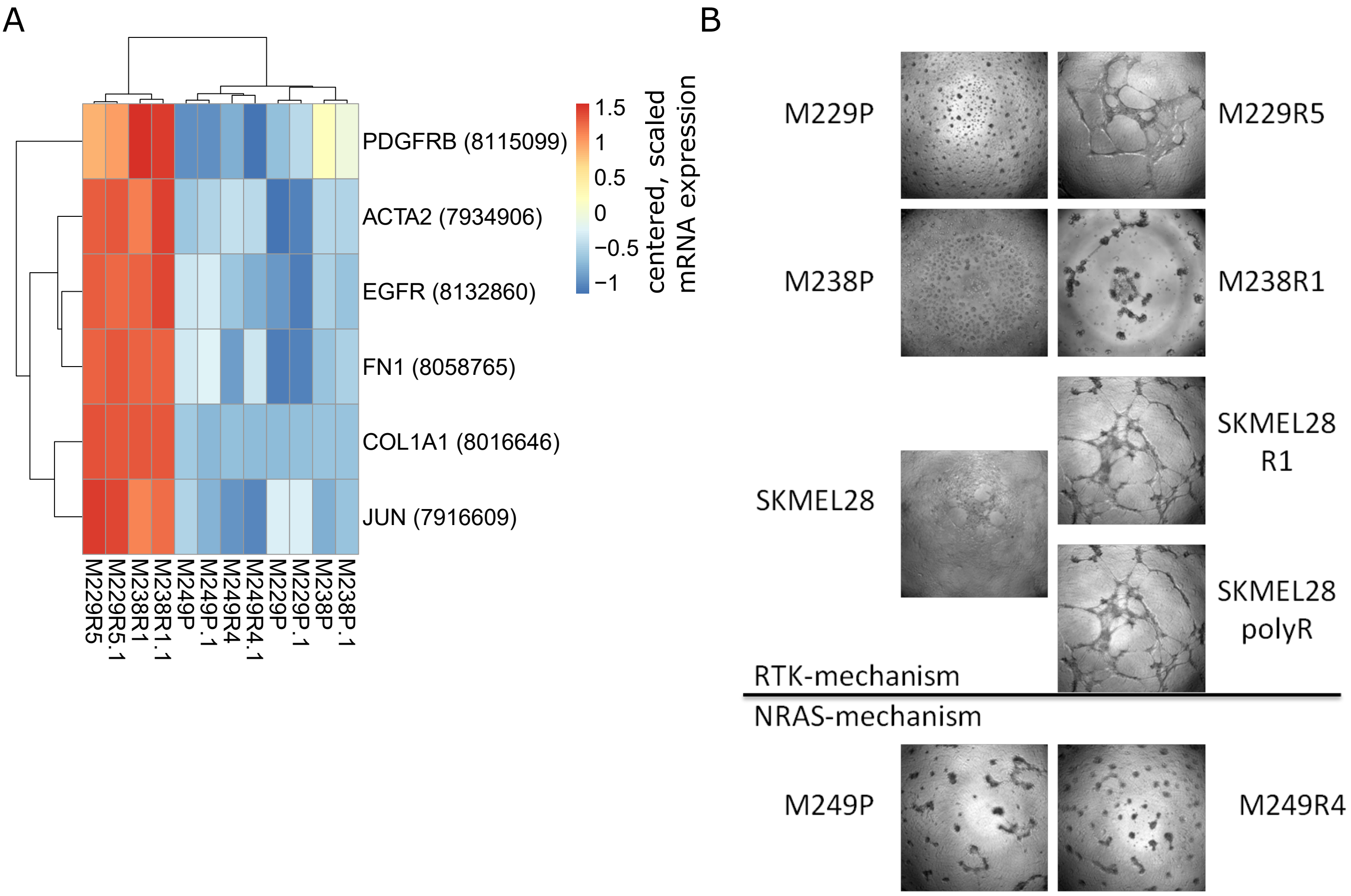

Comparison of phenotypes of RTK-resistance mechanism with NRAS-mechanism melanoma cells. (A) JUN, PDGFRB, EGFR, FN1, ACTA2, and COL1A1 mRNA expression for the RTK-resistance (M229 and M238) and NRAS-resistance (M249) parental/resistant cell line pairs. Microarray expression data from Nazarian et al. Expression values are centered and scaled for each gene. For genes represented by more than one probe set, the probe set with the largest absolute measured values was selected. (B) Comparison of tubuli-formation assay for three RTK-resistance (M229, M238, SKMEL28) and the NRAS-resistance (M249) parental/resistant cell lines. Note that both the M249 parental and resistant cell line shows more clumping, but no tubuli formation. Compare with Figure 3D.
