# Supplementary material for: JUN dependency in distinct early and late BRAF inhibition adaptation states of melanoma
Source: Cell Discov. 2016 Sep 6;2:16028–. doi: 10.1038/celldisc.2016.28 (PMC5012007; doi:10.1038/celldisc.2016.28)
Supplement: Supplementary Figure S5 [file celldisc201628-s6.pdf]

Titz et al. Supplementary Figure S5

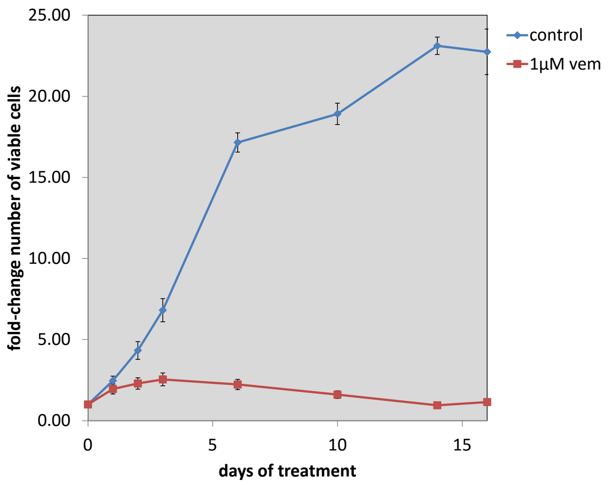

A cell sub-population persists under long-term vemurafenib treatment. M238P cells were treated for 16 days with 1µM vemurafenib or the control (media alone). The number of viable cells was monitored over time (ViCell counter). Error bars represent SEM from 3 replicates.
