# Supplementary figures and images for: JUN dependency in distinct early and late BRAF inhibition adaptation states of melanoma
Source: Cell Discov. 2016 Sep 6;2:16028–. doi: 10.1038/celldisc.2016.28 (PMC5012007; doi:10.1038/celldisc.2016.28)

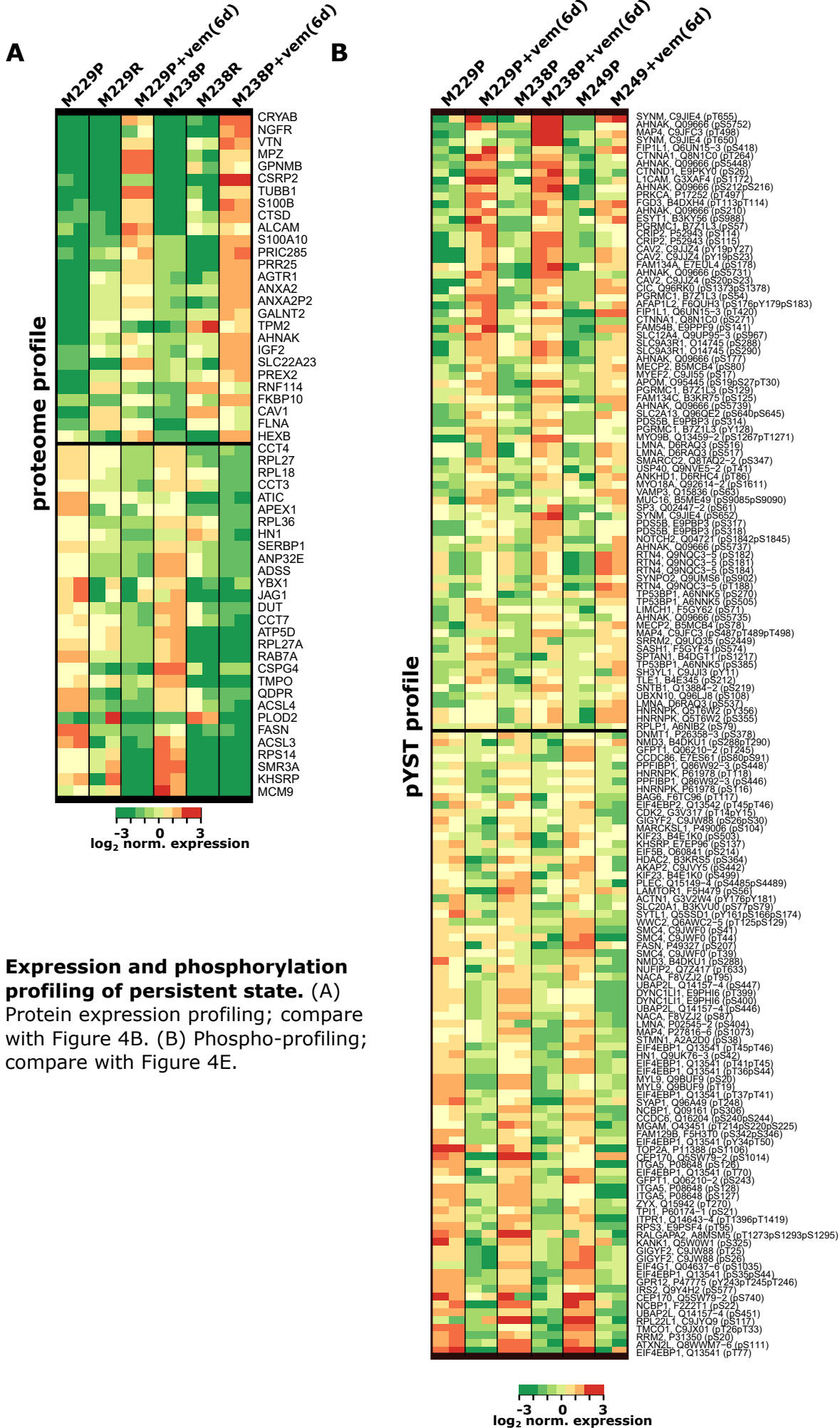

Supplement: Supplementary Figure S6 [file celldisc201628-s7.pdf]

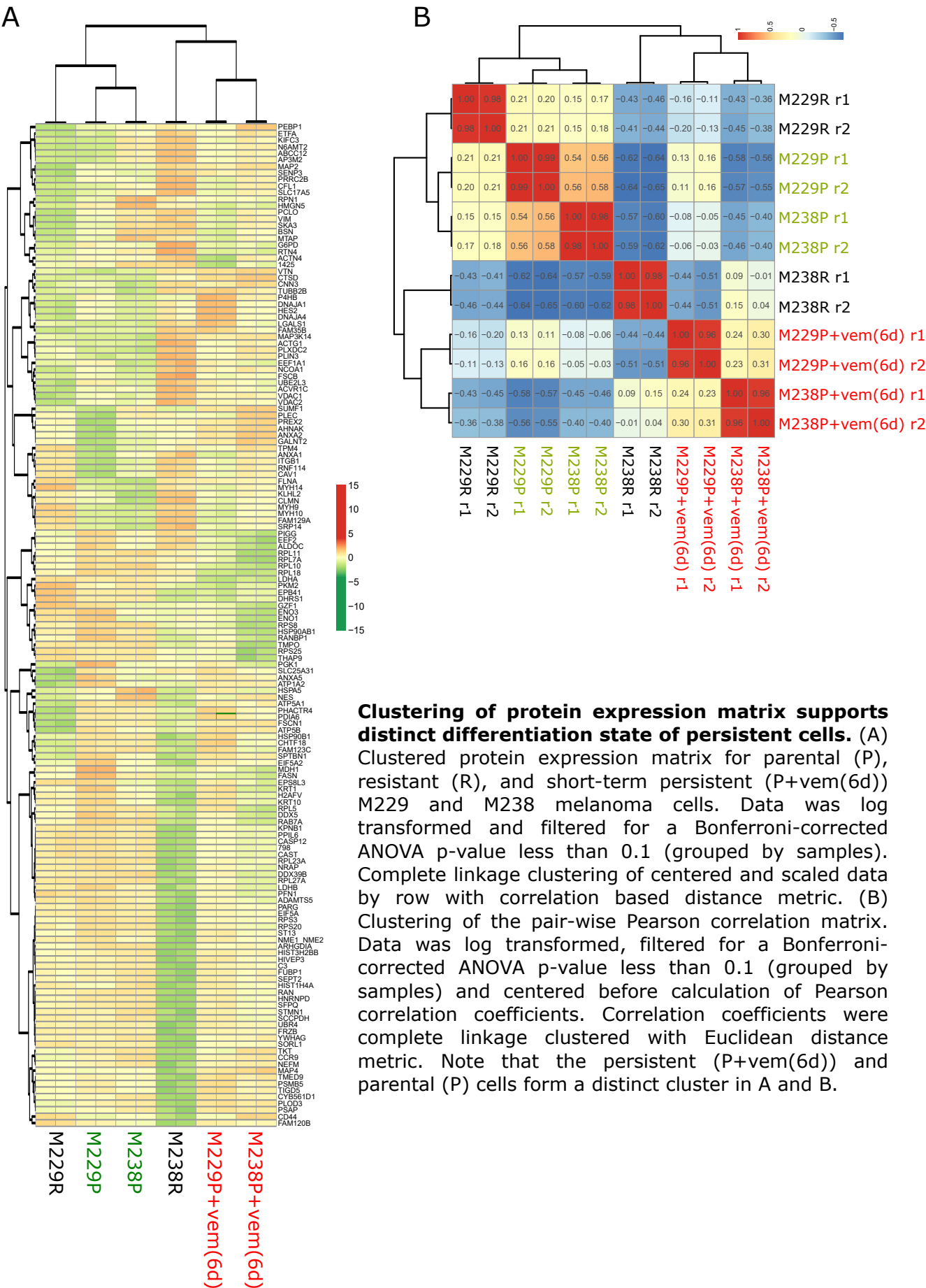

Supplement: Supplementary Figure S7 [file celldisc201628-s8.pdf]
