# Supplementary material for: JUN dependency in distinct early and late BRAF inhibition adaptation states of melanoma
Source: Cell Discov. 2016 Sep 6;2:16028–. doi: 10.1038/celldisc.2016.28 (PMC5012007; doi:10.1038/celldisc.2016.28)
Supplement: Supplementary Figure S8 [file celldisc201628-s9.pdf]

Titz et al. Supplementary Figure S8

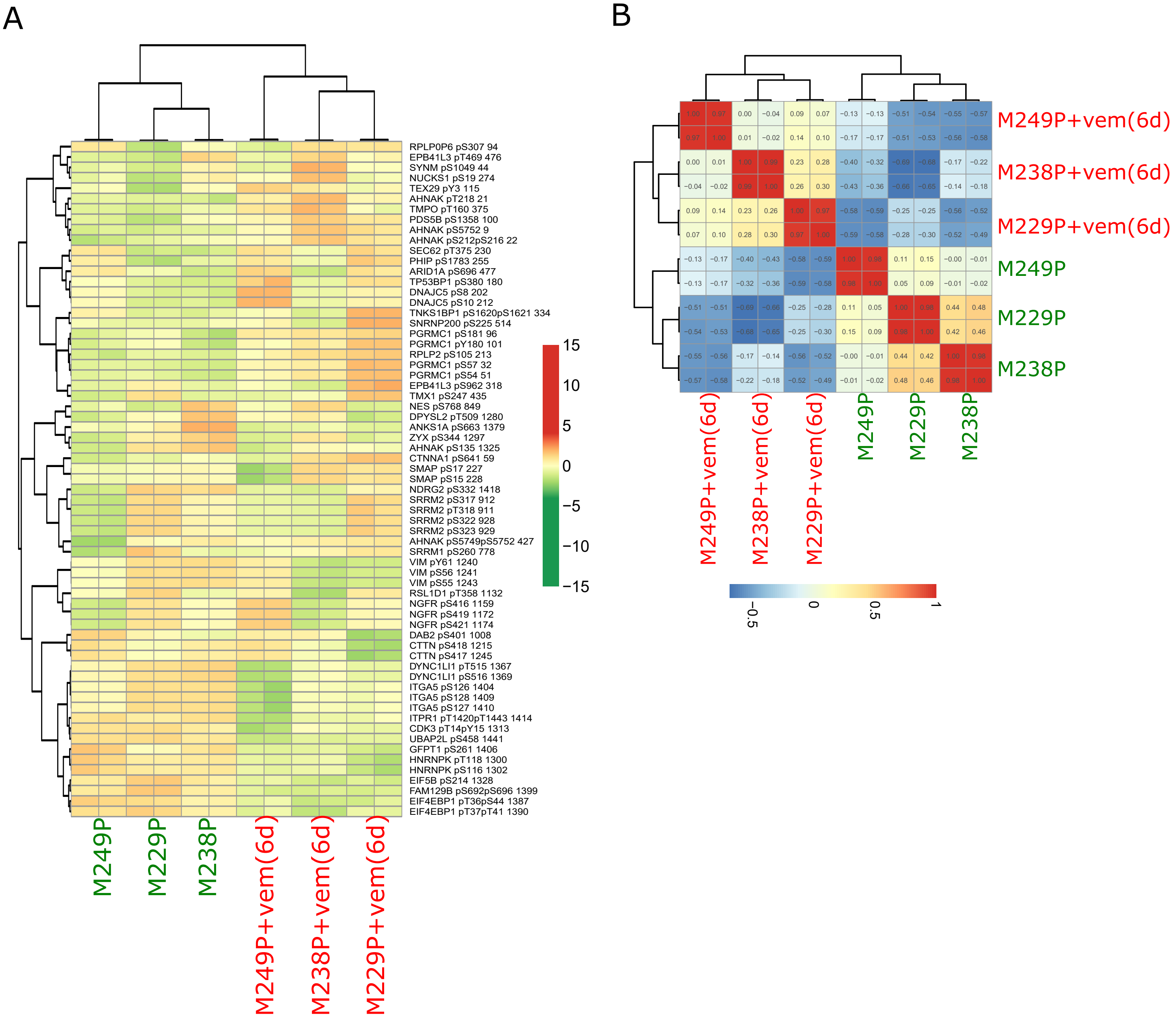

Clustering of phosphorylation site abundance matrix supports distinct signaling state of persistent cells. (A) Clustered phosphorylation site abundance matrix for parental (P) and short-term persistent (S) M229, M238, and M249 melanoma cells. Quantile normalized data was log-transformed and filtered for a Bonferroni-corrected ANOVA p-value less than 0.1 (grouped by samples). Complete linkage clustering of centered and scaled data by row with correlation based distance metric. (B) Clustering of the pair-wise Pearson correlation matrix. Quantile normalized data was log transformed, filtered for a Bonferroni-corrected ANOVA p-value less than 0.1 (grouped by samples) and centered before calculation of Pearson correlation coefficients. Correlation coefficients were complete linkage clustered with Euclidean distance metric.
