# Supplementary material for: JUN dependency in distinct early and late BRAF inhibition adaptation states of melanoma
Source: Cell Discov. 2016 Sep 6;2:16028–. doi: 10.1038/celldisc.2016.28 (PMC5012007; doi:10.1038/celldisc.2016.28)
Supplement: Supplementary Figure S9 [file celldisc201628-s10.pdf]

Titz et al. Supplementary Figure S9

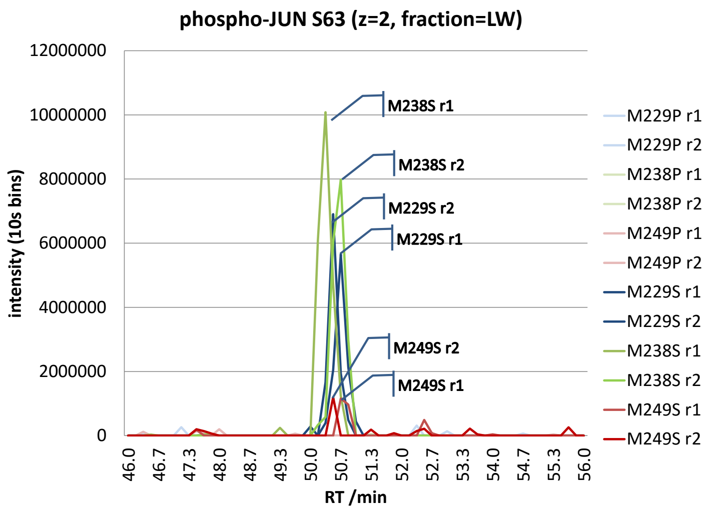

Persistent cells show higher expression of phospho-JUN S63 peptide. Selected extracted ion chromatogram shown for phospho-JUN S63 peptide in short-term vemurafenib phospho-profiling experiment (fraction LW, charge state (z) = 2).
