# Supplementary material for: JUN dependency in distinct early and late BRAF inhibition adaptation states of melanoma
Source: Cell Discov. 2016 Sep 6;2:16028–. doi: 10.1038/celldisc.2016.28 (PMC5012007; doi:10.1038/celldisc.2016.28)
Supplement: Supplementary Figure S10 [file celldisc201628-s11.pdf]

Titz et al. Supplementary Figure S10

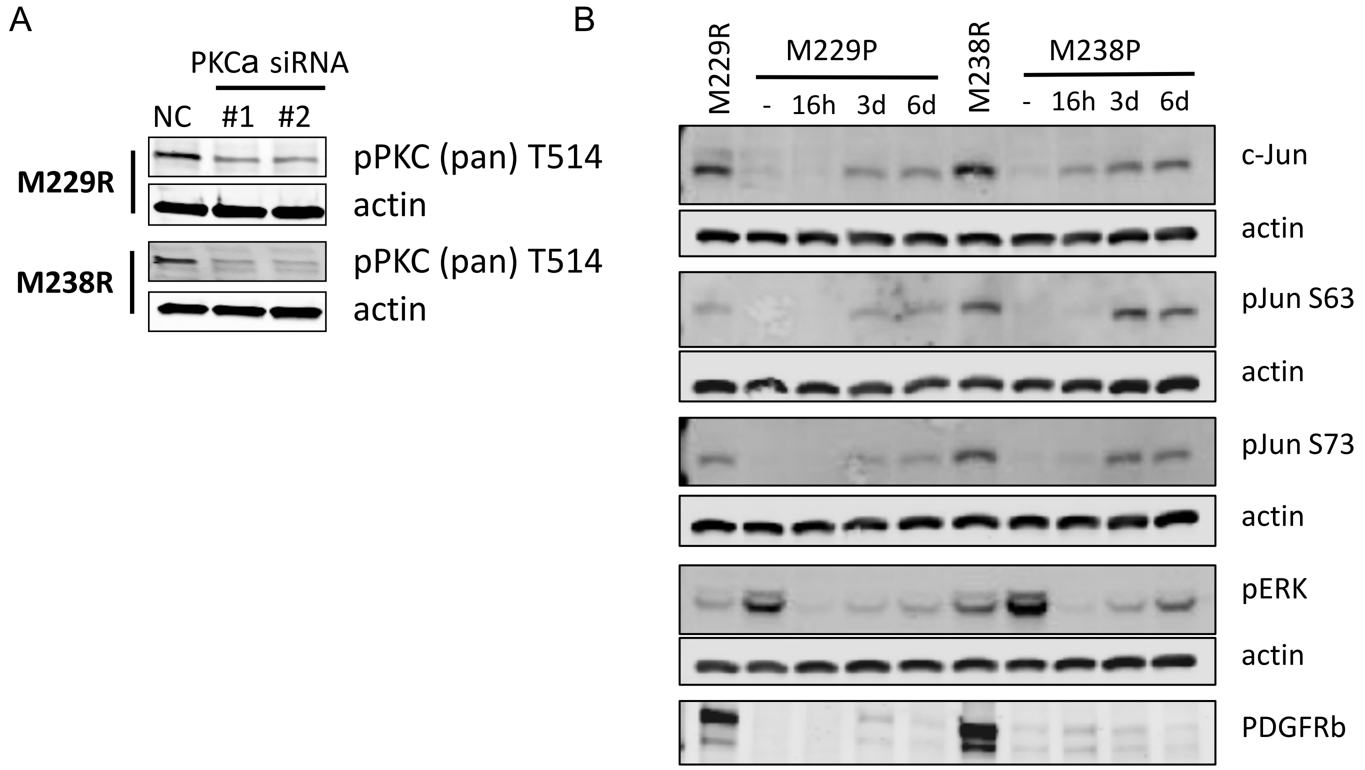

PKC isoform analysis and extended vemurafenib treatment kinetics. (A) The upregulated PKC isoform in M229R and M238R corresponds to PKC . The detected upregulated PKC isoform was successfully targeted with two independent PKC -specific siRNAs (#1 = GGGAUCUGAAGUUAGAUAATT, #2 = GCUGGUUAUUGCUAACAUATT, NC = negative scramble control, obtained from Shanghai GenePharma, Shanghai, China). (B) Extended kinetics compared to Figure 5E. M229P and M238P were left untreated (-) or treated with 1uM vemurafenib/PLX4032 (vem) for 3h, 16h, 3 days, and 6 days and compared by Western blot with the fully resistant cells (M229R and M238R).
