# Supplementary material for: JUN dependency in distinct early and late BRAF inhibition adaptation states of melanoma
Source: Cell Discov. 2016 Sep 6;2:16028–. doi: 10.1038/celldisc.2016.28 (PMC5012007; doi:10.1038/celldisc.2016.28)
Supplement: Supplementary Figure S11 [file celldisc201628-s12.pdf]

Titz et al. Supplementary Figure S11

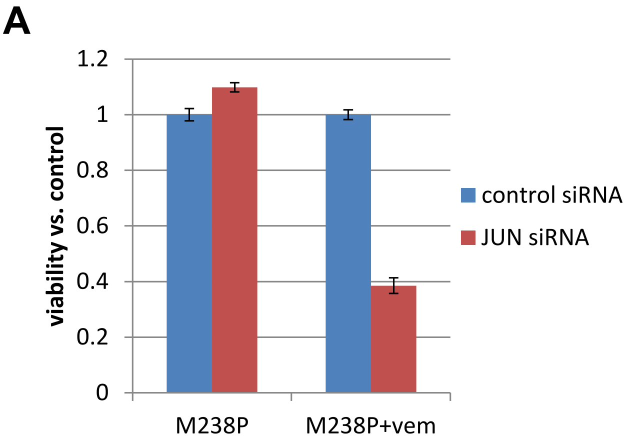

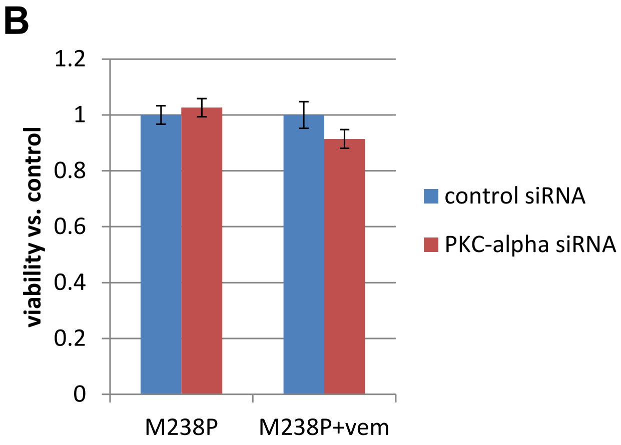

JUN knock-down not PKC-alpha knockdown is effective co-treatment option. Effect on cell viability of vemurafenib and JUN or PKC-alpha siRNA knockdown co-treatment for parental (M238P) and vemurafenib co-treated M238P cells (M238P+vem). Cell viability was assessed after 3 days by a MTS assay. (A) JUN knockdown. (B) PKC-alpha knockdown
