# Supplementary material for: JUN dependency in distinct early and late BRAF inhibition adaptation states of melanoma
Source: Cell Discov. 2016 Sep 6;2:16028–. doi: 10.1038/celldisc.2016.28 (PMC5012007; doi:10.1038/celldisc.2016.28)
Supplement: Supplementary Figure S12 [file celldisc201628-s13.pdf]

Titz et al. Supplementary Figure S12

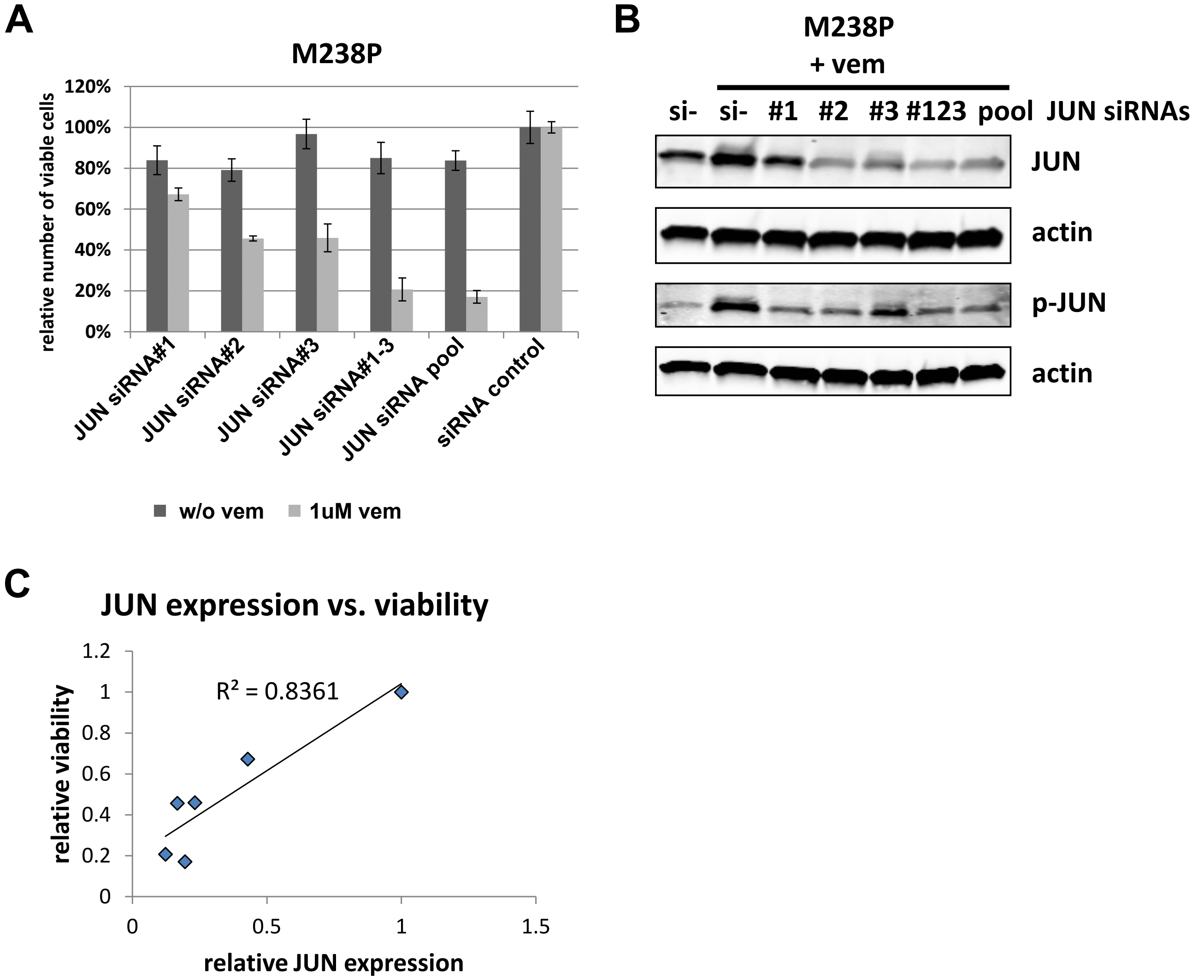

Combination of vemurafenib and JUN knockdown with individual siRNAs. M238P cells were transfected with individual JUN siRNAs, a combination of three siRNAs (#1, #2, and #3), the full pool (siRNA #1-#4), or a control siRNA (si-). Transfected cells were kept without and with 1uM vemurafenib/PLX4032 (vem). (A) The effect on cell viability was assessed. Normalized viable cell number relative to control siRNA. (B) The effect on (phospho-) JUN expression was analyzed by Western blot. (C) Correlation of remaining JUN expression and relative viability of vemurafenib treated cell in A and B. JUN siRNAs obtained from Thermo Scientific: siGENOME SMARTpool siRNA D-003268-22 (#1), D-003268-08 (#2), D-003268-05 (#3), and D-003268-09 (#4).
