# Supplementary material for: JUN dependency in distinct early and late BRAF inhibition adaptation states of melanoma
Source: Cell Discov. 2016 Sep 6;2:16028–. doi: 10.1038/celldisc.2016.28 (PMC5012007; doi:10.1038/celldisc.2016.28)
Supplement: Supplementary Figure S13 [file celldisc201628-s14.pdf]

Titz et al. Supplementary Figure S13

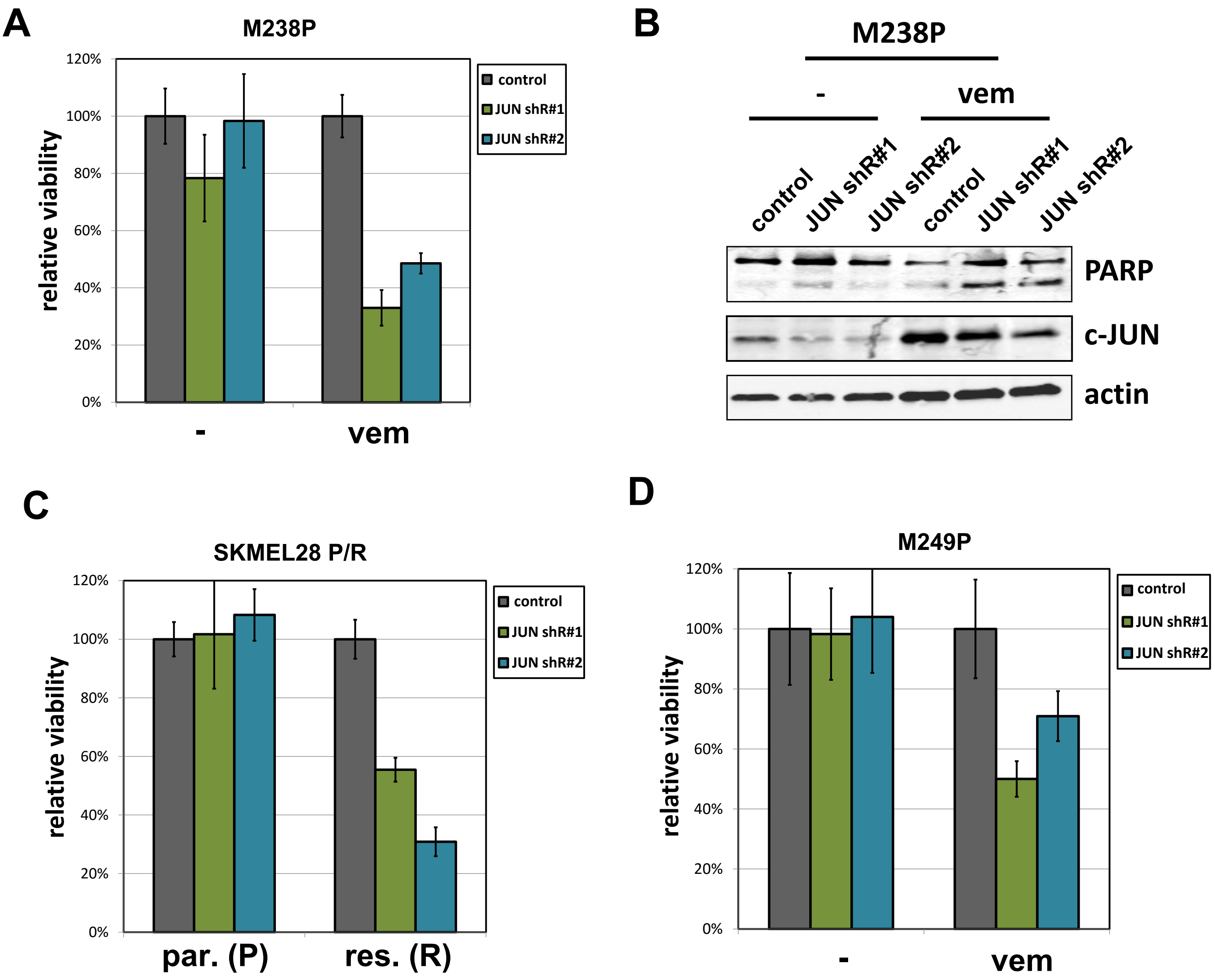

Combination of vemurafenib and shRNA-mediated JUN knockdown. (A) M238P cells were treated with (vem) and without (-) 1uM vemurafenib and either control shRNA or two different JUN shRNAs (as lentiviral particles, JUN shR#1 and #2). Relative viability to control shRNA measured by MTS assay. (B) Western blot of M238P cells treated as in A. Cleaved PARP shown as an apoptosis marker. (C) Relative number of viable cells for parental (P) and RTK-upregulated resistant (R) SKMEL28 cells. JUN shRNA treatment as in A; resistant cells cultured in presence of 1uM vemurafenib. (D) Relative number of viable cells for M249P cells mock treated (-) or treated with 1uM vemurafenib and JUN shRNAs. shRNA#1 corresponds to Mission shRNA TRCN0000010366 (Sigma, CCGGTAGTACTCCTTAAGAACA CAACTCGAGTTGTGTT CTTAAGGAG TACTATTTTTG) and shRNA#2 to shRNA TRCN0000039590.
