# Supplementary material for: JUN dependency in distinct early and late BRAF inhibition adaptation states of melanoma
Source: Cell Discov. 2016 Sep 6;2:16028–. doi: 10.1038/celldisc.2016.28 (PMC5012007; doi:10.1038/celldisc.2016.28)
Supplement: Supplementary Figure S14 [file celldisc201628-s15.pdf]

Titz et al. Supplementary Figure S14

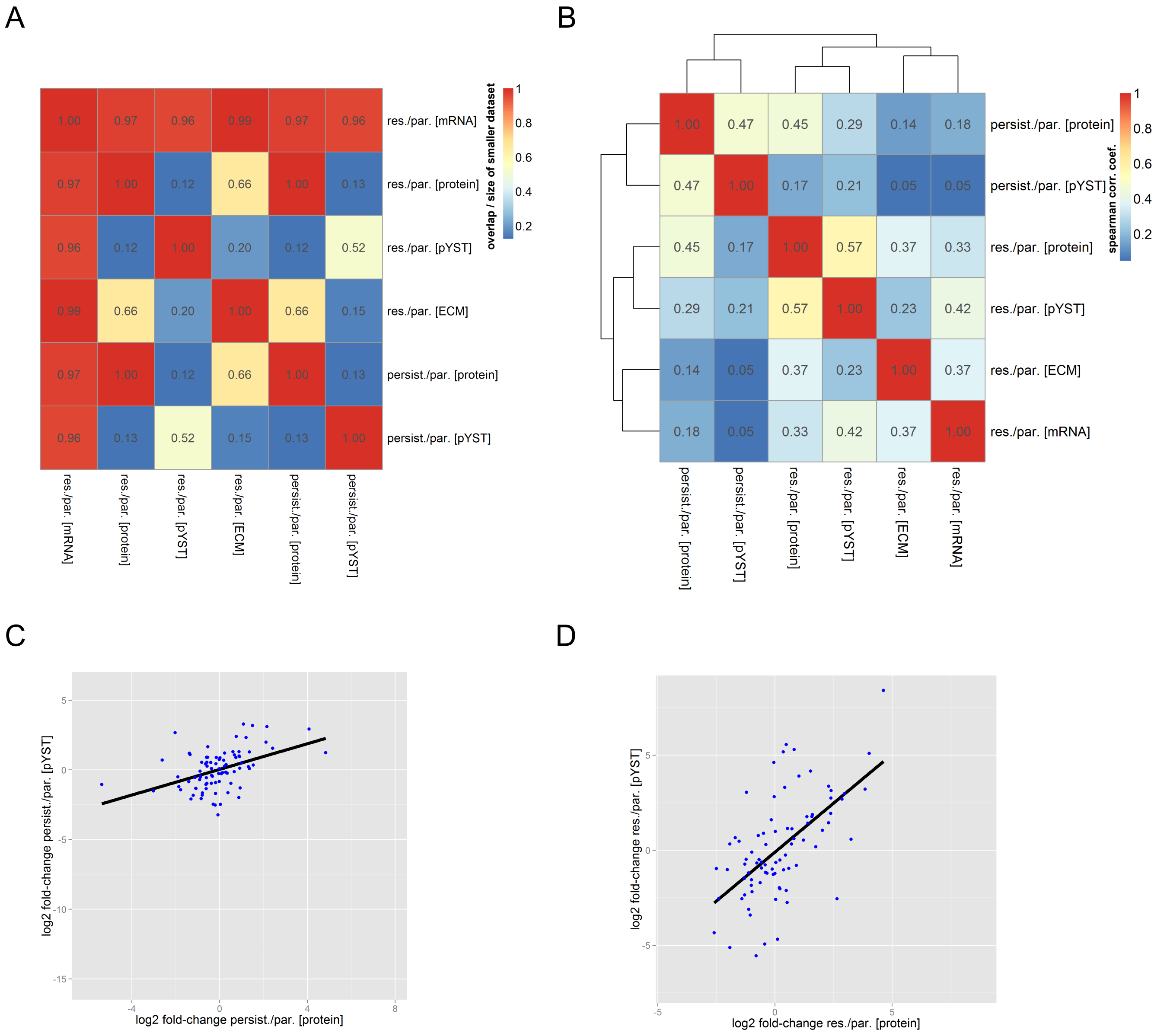

Comparison of the transcriptomics and proteomics datasets for parental, resistant, and persistant M229 and M238 cells. (A) Fraction of genes/proteins shared between two datasets, ie. number of detected proteins/genes in both studies divided by the number of genes/proteins in the study with lower coverage. mRNA = microarray expression dataset (Nazarian et al.); protein = total protein profiling; pYST = phospho-profiling; ECM = extracellular matrix profiling; res./par. = resistant/parental cell line pairs; persist./par. = persistent/parental cell line pairs. Note that the total protein expression and phospho-profiling datasets show only a limited fraction of proteins detected (and quantified) in both datasets (12% and 13%). (B) Clustered spearman correlation matrix comparing the different datasets. All M229 and M238 replicates were combined and average log2 fold-changes for resistant/parental and persistent/parental cells were calculated. Pair-wise spearman correlation coefficients (number in matrix) were calculated and the correlation matrix clustered by complete linkage clustering with a Euclidean distance metric. Note the (limited) similarity between the total protein and pYST fold-changes for both the persistent/parental and the resistant/parental paris. However, remember the limited number of shared proteins between these datasets. (C,D) Scatter plots showing the comparison of the total protein and pYST fold-changes for the persistent/parental (C) and the resistant/parental pairs (D). The linear regression line is shown in black.
