# Supplementary material for: JUN dependency in distinct early and late BRAF inhibition adaptation states of melanoma
Source: Cell Discov. 2016 Sep 6;2:16028–. doi: 10.1038/celldisc.2016.28 (PMC5012007; doi:10.1038/celldisc.2016.28)
Supplement: Supplementary Figure S15 [file celldisc201628-s16.pdf]

Titz et al. Supplementary Figure S15

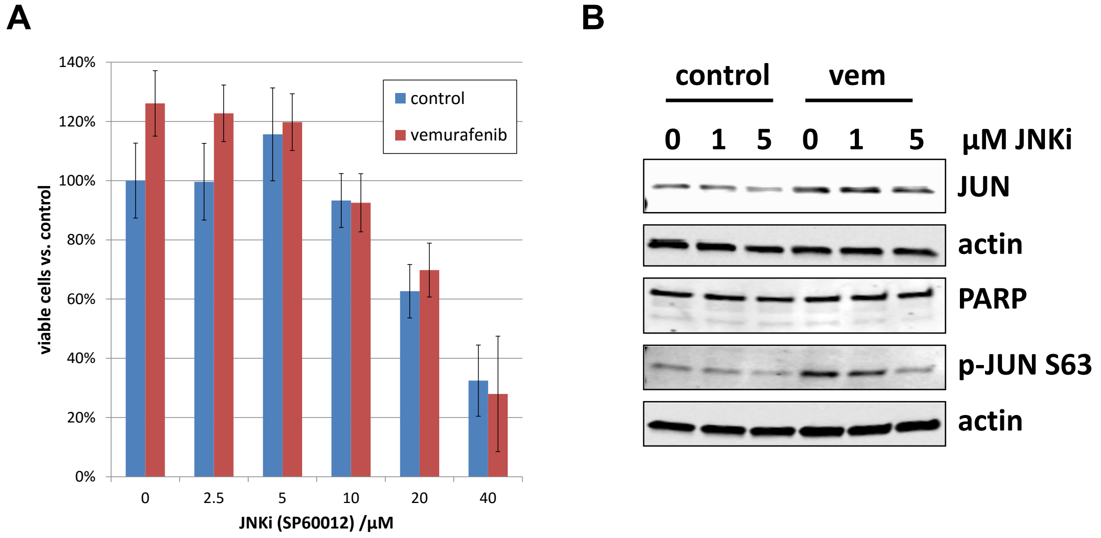

Pharmacological inhibition of c-Jun N-terminal kinases (JNKs) is ineffective at targeting the persistent state. (A) Combining JNK inhibitor (JNKi, SP600125) and vemurafenib treatment of M238P cells does not show synergistic or additive effect in viability experiment. Cells were pretreated with indicated concentration of JNKi (or DMSO control) and 1µM vemurafenib (or DMSO control). General toxicity of the JNK inhibitor (JNKi) is observed only above 10µM. (B) JNKi effectively suppresses JUN phosphorylation in M238P cells. Western blot of M238P cells treated with increasing concentrations of JNKi (0, 1, 5µM) and with (vem) or without (control) 1µM vemurafenib. Note that suppression of JUN phosphorylation is observed at lower concentration than for toxicity in (A).
